# Lytic polysaccharide monooxygenases: a crystallographer’s view on a new class of biomass-degrading enzymes

**DOI:** 10.1107/S2052252516014147

**Published:** 2016-10-14

**Authors:** Kristian E. H. Frandsen, Leila Lo Leggio

**Affiliations:** a Department of Chemistry, University of Copenhagen, Universitetsparken 5, DK-2100 Copenhagen, Denmark

**Keywords:** lytic polysaccharide monooxygenases, carbohydrate-modifying enzymes, metalloproteins, copper enzymes, biomass degradation

## Abstract

Lytic polysaccharide monooxygenases (LPMOs) are a new class of microbial copper enzymes involved in the degradation of recalcitrant polysaccharides. They have only been discovered and characterized in the last 5–10 years and have stimulated strong interest both in biotechnology and in bioinorganic chemistry. In biotechnology, the hope is that these enzymes will finally help to make enzymatic biomass conversion, especially of lignocellulosic plant waste, economically attractive. Here, the role of LPMOs is likely to be in attacking bonds that are not accessible to other enzymes. LPMOs have attracted enormous interest since their discovery. The emphasis in this review is on the past and present contribution of crystallographic studies as a guide to functional understanding, with a final look towards the future.

## Abstract 2

Lytic polysaccharide monooxygenases (LPMOs) are a new class of metalloenzymes discovered in the last decade. LPMOs are now thought to be key enzymes in the biological and biotechnological degradation of biomass and are reviewed here from a structural biology viewpoint.

## 1. Discovery of LMPOs and initial structural and functional studies   

### 1.1. Setting the scene: enzymes breaking glycosidic linkages   

**Figure 1:**
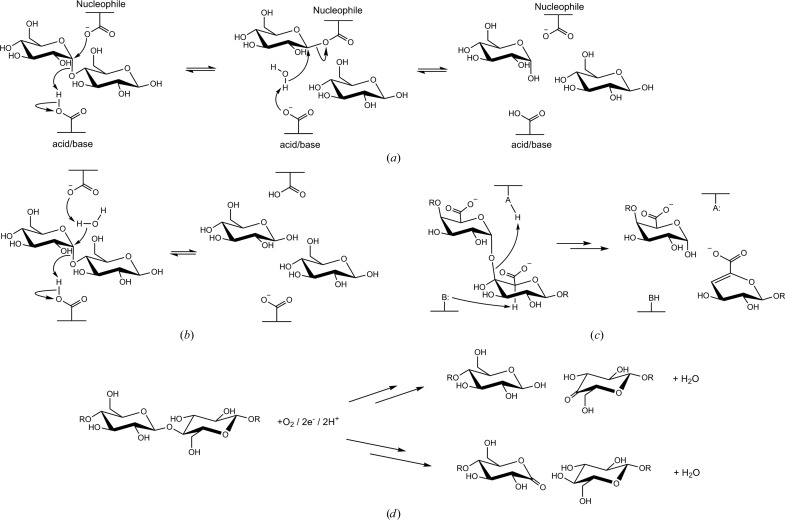
Enzymatic strategies for cleavage of glycosidic linkages. Glycoside hydrolysis of maltose by a retaining (*a*) or inverting (*b*) mechanism, polygalacturonan degradation by a polysaccharide lyase (*c*) and oxidative cleavage of cellooligosaccharides/cellulose by LPMOs (*d*).

Enzymes that are able to break glycosidic linkages (which we refer to here generically as glycosidases) have been of great interest to scientists for about a century. Alexander Fleming discovered the antibacterial properties of mucus lysozyme (Fleming, 1922 ▸) in his quest for antibiotics. Lysozyme acts as an antibacterial by cleaving the glycosidic linkage in bacterial peptidoglycans and has since become one of the most important models in protein chemistry. The hen egg-white variant was the first enzyme for which a high-resolution structure was determined, by David C. Phillips in the 1960s (Blake *et al.*, 1965 ▸), paving the way for the understanding of enzyme mechanisms at the atomic level. Influenza neuraminidase is another example of a glycosidase which is essential for the release of virus particles from infected cells, and has been a major structure-based drug-design target (von Itzstein & Thomson, 2009 ▸). Lysozyme and neuraminidase have in common a hydrolytic mechanism for breaking the glycosidic linkage, which they share with most known glycosidases and for which examples are shown in Figs. 1 ▸(*a*) and 1 ▸(*b*). An alternative mechanism for breaking the glycosidic linkage introduces a carbon–carbon double bond in the product and is used by, for example, the plant pathogen virulence factors known as pectate lyases (Yoder *et al.*, 1993 ▸; Fig. 1 ▸ *c*).

Glycoactive enzymes have also attracted great interest for the exploitation of biomass. Biomass from land plants (Bornscheuer *et al.*, 2014 ▸), algae (Wei *et al.*, 2013 ▸), insects and crustacean shells (Hayes *et al.*, 2008 ▸) is rich in polysaccharides. Instead of accumulating in landfills or being burnt, waste biomass could be exploited for the production of bio­ethanol or value-added products such as biodegradable plastics, sweeteners, pharmaceuticals *etc.* (Bayer *et al.*, 2007 ▸; Bornscheuer *et al.*, 2014 ▸; Vaaje-Kolstad *et al.*, 2013 ▸). However, depolymerization of the polysaccharides into fermentable sugars or simpler building blocks is necessary for most applications, but is hindered by crystallinity and the complex matrix in which the polysaccharides are embedded (Carpita & Gibeaut, 1993 ▸; Zeng *et al.*, 2014 ▸). Consequently, considerable efforts have been made in the study of microbial cellulose, hemicelluloses and chitin-degrading enzymes. A classification system for cellulases/xylanases (Henrissat *et al.*, 1989 ▸) was in fact one of the predecessors of the the CAZy (Carbohydrate Active enZYmes) database (Henrissat, 1991 ▸; Lombard *et al.*, 2014 ▸), which is arguably the most useful bioinformatics resource in glycobiology. The CAZy database is sequence- and structure-based, and thus can easily incorporate genomic data, yet tries to make the connection to function wherever possible. CAZy currently classifies glycoside hydrolases into GH families 1–135, and many more carbohydrate-active enzymes into other families.

### 1.2. The early history of LPMO discovery   

The first two families of LPMOs to be discovered were a fungal and mainly cellulolytic family and a mainly bacterial and chitinolyic family. The somewhat independent early histories of these two families, which eventually converged in 2010, are summarized here, but are outlined in more detail in at least two previous reviews (Lo Leggio *et al.*, 2012 ▸; Vaaje-Kolstad, Horn *et al.*, 2005 ▸). CBP21 (chitin-binding protein 21) was the first LPMO to be characterized in detail as part of the chitinolytic system of the bacterium *Serratia marcescens* (Vaaje-Kolstad *et al.*, 2013 ▸). Identified in 1986 (Fuchs *et al.*, 1986 ▸), it was first thought to have the main function of chitin binding (Suzuki *et al.*, 1998 ▸), and as such was classified into a family of carbohydrate-binding modules (CBMs; Boraston *et al.*, 2004 ▸) in the CAZy database (CBM33). The structure was determined in 2005 (Vaaje-Kolstad, Houston *et al.*, 2005 ▸; see below) and on first inspection seemed consistent with the proposed chitin-binding role. In the same year, it was established that CBP21 acted synergistically with chitin-active glycoside hydrolases to boost chitin degradation (Vaaje-Kolstad *et al.*, 2013 ▸). The oxidoreductase activity was first discovered in 2010 (Vaaje-Kolstad *et al.*, 2010 ▸), finally assigning the proper enzymatic role to CBP21 and other proteins belonging to CBM33.

In parallel to the discovery of these bacterial chitinolytic LPMOs, a fungal family of cellulolytic enzymes, initially classified as glycoside hydrolases in GH61, was puzzling researchers in the field. The family was reported in the literature in 1997 (Henrissat & Davies, 1997 ▸) and had just four members in 2001 (Karlsson *et al.*, 2001 ▸). Despite strong implication in cellulose degradation, demonstration of cellulo­lytic activity was problematic, as reviewed in Lo Leggio *et al.* (2012 ▸). In 2006 a patent indicated that GH61 could act synergistically to boost conventional cellulolytic hydrolases (Brown *et al.*, 2006 ▸). The first structures (see §[Sec sec2.1]2.1 for further details; Karkehabadi *et al.*, 2008 ▸; Welner *et al.*, 2009 ▸; Harris *et al.*, 2010 ▸) firmly established the structural similarity to CBP21, and furthermore revealed the presence of divalent metal ions at a structurally conserved site (see §[Sec sec2.2]2.2), which had not been clear in the first CBP21 structure. In 2010, the same year as in which enzymatic activity of CBP21 was demonstrated, additional evidence of the synergy between GH61 and cellulose-degrading glycoside hydrolases was presented, together with structure-based mutagenesis of the metal site which linked it to activity (Harris *et al.*, 2010 ▸). Thus, 2010 really marks the beginning of the systematic study of LPMOs as major factors in the degradation of recalcitrant polysaccharides, although oxidative action was first demonstrated for GH61 in 2011 (Quinlan *et al.*, 2011 ▸; Phillips *et al.*, 2011 ▸; Westereng *et al.*, 2011 ▸; Langston *et al.*, 2011 ▸).

LPMOs introduce a single O atom from molecular oxygen into the product, and utilize an external electron donor, for example ascorbate, in the process (Fig. 1 ▸ *d*; Vaaje-Kolstad *et al.*, 2010 ▸). It is remarkable that although the importance of redox chemistry in the degradation of cellulose has been recognized since at least the 1970s (Eriksson *et al.*, 1974 ▸), LPMO activity was first proved 40 years later, revolutionizing our previous view of cellulose and biomass degradation in nature (Béguin, 1990 ▸). It must be emphasized that the involvement of redox enzymes in biomass degradation, in itself, is not novel. Lignin degradation in particular, although as yet rather poorly characterized, is known to rely heavily on redox enzymes such as peroxidases and laccases (Guerriero *et al.*, 2016 ▸; Cragg *et al.*, 2015 ▸; Pollegioni *et al.*, 2015 ▸). Carbohydrate oxidases that oxidize monosaccharides, disaccharides and oligosaccharides (van Hellemond *et al.*, 2006 ▸), but without leading to chain cleavage, have also been known for a number of years, and their biological functions are varied and often still rather unclear, although lignocellulose degradation is also one of them. Lignin-degrading enzymes are not always very specific for their substrate, and often generate reactive species that can dissociate from the enzyme active site and act distally on a number of substrates, while some of the carbohydrate oxidases that have long been implicated in lignocellulose degradation act in indirect ways, for example by producing peroxide equivalents for other lignin-degrading enzymes. What is truly novel for LPMOs as redox enzymes in biomass degradation is their implication in the direct and specific depolymerization of polysaccharides, a biological function that was previously thought to be almost exclusively performed by hydrolases. This discovery has had far-reaching consequences for biotechnological applications and our understanding of the carbon cycle in nature (see also §[Sec sec1.3]1.3).

These discoveries prompted the reclassification of LPMOs in CAZy as auxiliary activities (AAs), together with other redox enzymes acting on lignin/lignocellulose, including many carbohydrate oxidases (Levasseur *et al.*, 2013 ▸). GH61 was renamed AA9, CBM33 was renamed AA10 and two additional LPMO families since identified have been named AA11 and AA13 (Hemsworth *et al.*, 2014 ▸; Vu, Beeson, Span *et al.*, 2014 ▸). From now on in this review we will indicate individual LPMOs by the initials of the Latin name of the organism in italics followed by the AA family and if necessary a further specifier, *e.g. Ta*AA9_A and *Sm*AA10_A (CBP21). Based on sequence, AA9 was further divided into groups suggested to reflect the site of oxidation. Thus, AA9 is subdivided into type 1 (C1-oxidizing), type 2 (C4-oxidizing) or type 3 (more promiscuous, generally C1- and C4-oxidizing, with the exception of PMO-3* which only oxidizes C1) (Vu, Beeson, Phillips *et al.*, 2014 ▸; Li *et al.*, 2012 ▸; Phillips *et al.*, 2011 ▸), but there is some controversy as to whether the oxidation site strictly follows a phylogenetic relationship. Other subdivisions of AA9 and other AA families based on sequence have been suggested, for example, by Busk & Lange (2015 ▸) and Book *et al.* (2014 ▸).

The importance of structural studies in the early stages of LPMO discovery cannot be overestimated, as they were instrumental in establishing that AA9 and AA10 were functionally linked and that their action, enzymatic or otherwise, was dependent on the presence of a metal. In the case of LPMOs structural knowledge really can claim to have driven functional understanding.

### 1.3. LPMOs: why all the fuss?   

As outlined above, LPMOs are, in a nutshell, a newly discovered class of oxidative copper enzymes that degrade polysaccharides, a previously unknown function for redox enzymes involved in biomass degradation. Since their discovery the literature regarding LPMOs has really taken off. A search for ‘polysaccharide monooxygenase’, ‘CBM33’, ‘GH61’, ‘CBP21’ and related terms in Web of Science (excluding patents) returned one relevant result in 2000, two in 2005, two in 2010 and 43 in 2015, with no sign of diminishing interest in 2016.

Before embarking on a detailed view of their structures, we would like to highlight a few of the reasons why these LPMOs have created such a stir. In addition to their potential in biomass degradation, which is perhaps the aspect that has attracted the most attention (Horn *et al.*, 2012 ▸; Harris *et al.*, 2014 ▸; Johansen, 2016*a* ▸), a medical dimension may well reveal itself to be very important in the future, since a number of bacterial chitinolytic systems have been implicated in virulence and pathogenicity (Frederiksen *et al.*, 2013 ▸). *Vibrio cholerae*, *Listeria monocytogenes* and *Enterococcus faecalis*, all of which are human pathogens, possess chitinolytic systems including an active AA10 LPMO (Loose *et al.*, 2014 ▸; Paspaliari *et al.*, 2015 ▸; Vaaje-Kolstad *et al.*, 2012 ▸).

LPMOs may have profound environmental impact in nature owing to their effect in the global carbon cycle (C cycle). Fungal species play a significant role in the turnover of terrestrial C pools and thus in the global C cycle (Glass *et al.*, 2013 ▸; Rytioja *et al.*, 2014 ▸; Floudas *et al.*, 2015 ▸). Fungi are often divided into either saprotrophs (degrading dead organic matter) or biotrophs with a symbiotic lifestyle with a plant host (mutualistic or parasitic). Saprotrophic filamentous fungi have a variety of plant cell wall-degrading enzymes, and are important for the turnover of carbon as they deconstruct lignocellulosic biomass. LPMO-encoding genes are highly abundant in these organisms and LPMOs are predicted to play a significant role in global carbon flux. In many symbiotic biotrophic fungi (known as mycorrhizal fungi) the number of genes encoding plant cell wall-degrading enzymes is greatly reduced (Kohler *et al.*, 2015 ▸; Shah *et al.*, 2016 ▸). However, several genes encoding lignocellulosic degrading oxidative enzymes are retained, of which a substantial part are LPMOs, indicating that they are also advantageous to biotrophic fungi, and thus expanding the importance of LPMOs in the natural C cycle. The abundance of LPMOs in nature has additional environmental consequences that are of interest to human life. AA9 LPMOs are well represented in plant fungal pathogens, where they may be factors in pathogenesis (Gibson *et al.*, 2011 ▸), and in fungi causing wood decay, especially white-rot fungi (Floudas *et al.*, 2012 ▸). Recently, fusolin, the protein that forms the spindles of insect poxviruses, which have potential in pest control, was found to consist of an AA10 LPMO (Chiu *et al.*, 2015 ▸).

The bioinorganic chemistry of LPMOs is also unusual and has attracted considerable attention: they have a type 2 copper site (Crichton, 2012 ▸), which will be reviewed in more detail in §[Sec sec2.2]2.2, but with only two histidines, whereas other enzymes have three or four. Furthermore, it is intriguing how a mononuclear copper site can achieve a two-electron reaction, which is one of the most supported current mechanistic hypotheses, and how it can break the extremely strong C—H bond (Walton & Davies, 2016 ▸).

Given the level of interest in LPMOs, a number of reviews have been written focusing solely or largely on these enzymes. Among the slightly older but still influential reviews are one focused on bioethanol production (Horn *et al.*, 2012 ▸) and one on early structural studies (Hemsworth, Davies *et al.*, 2013 ▸). Most recent reviews have covered specific aspects such as the important role of LPMOs in lignocellulose degradation across the tree of life (Cragg *et al.*, 2015 ▸), their biotechnological potential (Hemsworth *et al.*, 2015 ▸), their mechanism (Walton & Davies, 2016 ▸), their industrial applications (Johansen, 2016*a* ▸) and their role in plant–microbe interactions (Johansen, 2016*b* ▸). Recently, a brief structural and functional overview of all LPMO families has also been published (Span & Marletta, 2015 ▸), as well as two more specific and detailed reviews on cellulose-degrading LPMOs (Beeson *et al.*, 2015 ▸) and starch-degrading LPMOs (Vu & Marletta, 2016 ▸). The present review attempts to be somewhat different from previous reviews, being written as it were by crystallographers with crystallo­graphers and other structural biologists as an audience, and not necessarily experts on LPMOs or carbohydrate-modifying enzymes. Furthermore, as the field moves extremely rapidly, we also hope to provide a useful update and overview for LPMO aficionados.

## 2. Structure-driven discovery of function   

### 2.1. First structures: the early years 2008–2010   

**Figure 2:**
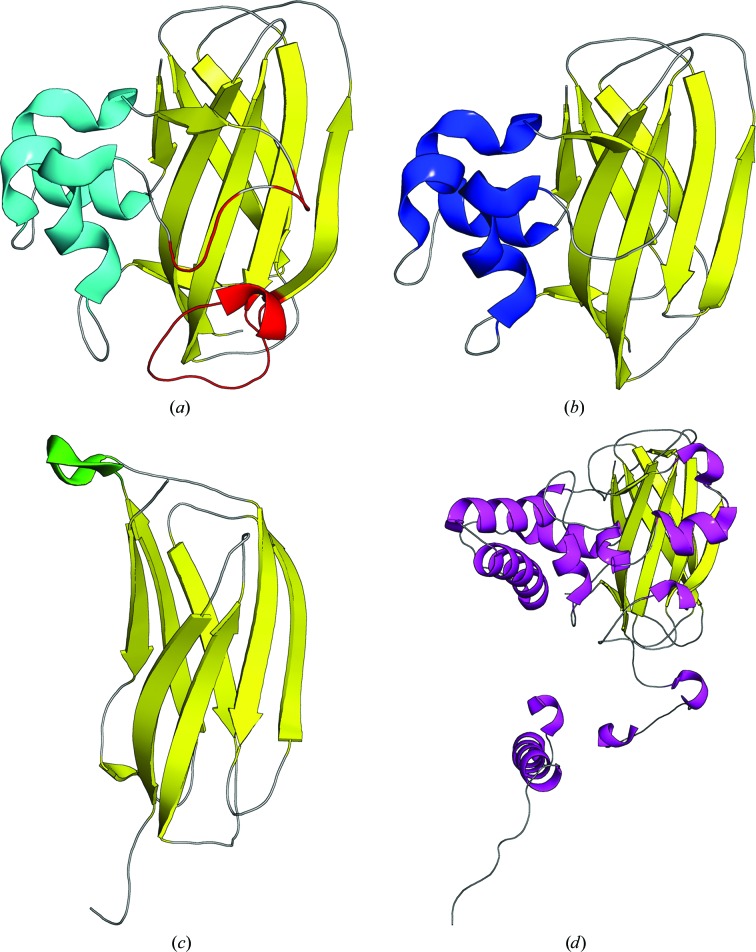
Overall view of selected AA10 structures and the closest non-LPMO structural relative. The structurally common central β-cores are coloured yellow, while distinct structural elements are coloured differently for each structure. (*a*) *Sm*AA10_A with the (‘budded’) helical insert in cyan and elements differing compared with *Jd*AA10_A indicated in red. (*b*) *Jd*AA10_A with the helical insert in blue. (*c*) The closest non-LPMO structural homologue (the MG2 domain of human α_2_-­macroglobulin; PDB entry 2p9r) with a small helical segment in green. (*d*) Fusolin (*Melolontha melolontha entomopoxvirus*; PDB entry 4ow5) with a number of helical segments in magenta.

It would be unfair to say that only structural knowledge has provided clues to the function of this class of proteins, but it has definitely played an enormous role. *Sm*AA10_A was the first LPMO for which a structure was determined (Vaaje-Kolstad, Houston *et al.*, 2005 ▸; please refer to Table 1 ▸ for the PDB codes and details of all structures that are mentioned). The structure of *Sm*AA10_A revealed a β-sandwich fold, described as a ‘budded’ fibronectin type III fold, where the bud consists of a 65-residue, predominantly helical insert between β-strands 1 and 2. The β-sandwich itself is rather unremarkable; a recent search of the Protein Data Bank (PDB) with *DALI* (Holm *et al.*, 2008 ▸) found PDB entry 2p9r (the MG2 domain of human α_2_-macroglobulin; Doan & Gettins, 2007 ▸) as the closest non-LPMO hit, and the backbone fit is remarkable (Figs. 2 ▸ *a* and 2 ▸ *c*) despite the absence of an obvious functional relationship. Surprisingly, the conserved aromatic residues that had been proposed to play a role in substrate binding prior to structure determination were instead found to form the hydrophobic core. The structure was key in identifying a patch of conserved and in part hydrophilic residues, and the role of most of these residues in substrate binding (especially Tyr45 and Glu60) was confirmed by mutagenesis. At this point, however, there was no suspicion that this could be a metalloenzyme and no metal site was identified.

When the first structures of fungal LPMOs in the AA9 family were determined, the most important discovery was perhaps their similarity to the structure of *Sm*AA10_A (Karkehabadi *et al.*, 2008 ▸; Welner *et al.*, 2009 ▸; Harris *et al.*, 2010 ▸), forming a definite connection between GH61 and CBM33 (now AA9 and AA10). Furthermore, the structures revealed unusual features for glycoside hydrolases; for example, the lack of a conserved carboxylate pair and a clear active-site cleft or groove. However, *Tt*AA9_E showed an arrangement of aromatic residues that was strongly reminiscent of a family 1 CBM, a type A CBM (meaning that it is specific for crystalline polysaccharides; Boraston *et al.*, 2004 ▸), and suggesting that these proteins acted by binding to crystalline cellulose.

Importantly, the structures also revealed a metal-binding site on the same face as these aromatic residues. This site was occupied by a nickel ion from the crystallization conditions (see Table 1 ▸) in the structure of *Trichoderma reesei* AA9_B (*Tr*AA9_B), which was actually determined by SAD using the nickel anomalous signal from data collected at a wavelength of 1.485 Å. The structure of *Tt*AA9_E, which shares only 29% sequence identity with *Tr*AA9_B and was determined by MIR (Welner *et al.*, 2009 ▸; Harris *et al.*, 2010 ▸), shows a similar metal-binding site. In the two deposited structures of *Tt*AA9_E the metal is modelled as an Mg or a Zn ion, depending on the crystallization/soaking conditions (Table 1 ▸). In retrospect, it is most likely that the metal visible in the ‘Mg’ complex is in fact a poorly occupied Cu ion; indeed, the *CheckMyMetal* server (Zheng *et al.*, 2014 ▸), which was unfortunately not yet available at the time, flags one of the ‘Mg’ sites as a possible copper-binding site. Arguably the major finding by Harris *et al.* (2010 ▸), comes from connecting the identified structural features to function by structure-guided mutagenesis of the relevant residues. Despite the limitations of the assay, which measured the boosting of conventional hydrolase activity, but without controlled inclusion of an electron donor or the correct metal, the studies demonstrated that the two His residues liganding the metal (one also through the N-terminus) were essential for activity, while a neighbouring Tyr and a conserved Gln which holds it in position by hydrogen bonding were almost essential. All of these residues are very highly conserved in AA9 sequences, as had already been noted by Karkehabadi *et al.* (2008 ▸). Furthermore, one of the Tyr residues in the putative cellulose-binding site was also important for activity.

### 2.2. The metal site   

**Figure 3:**
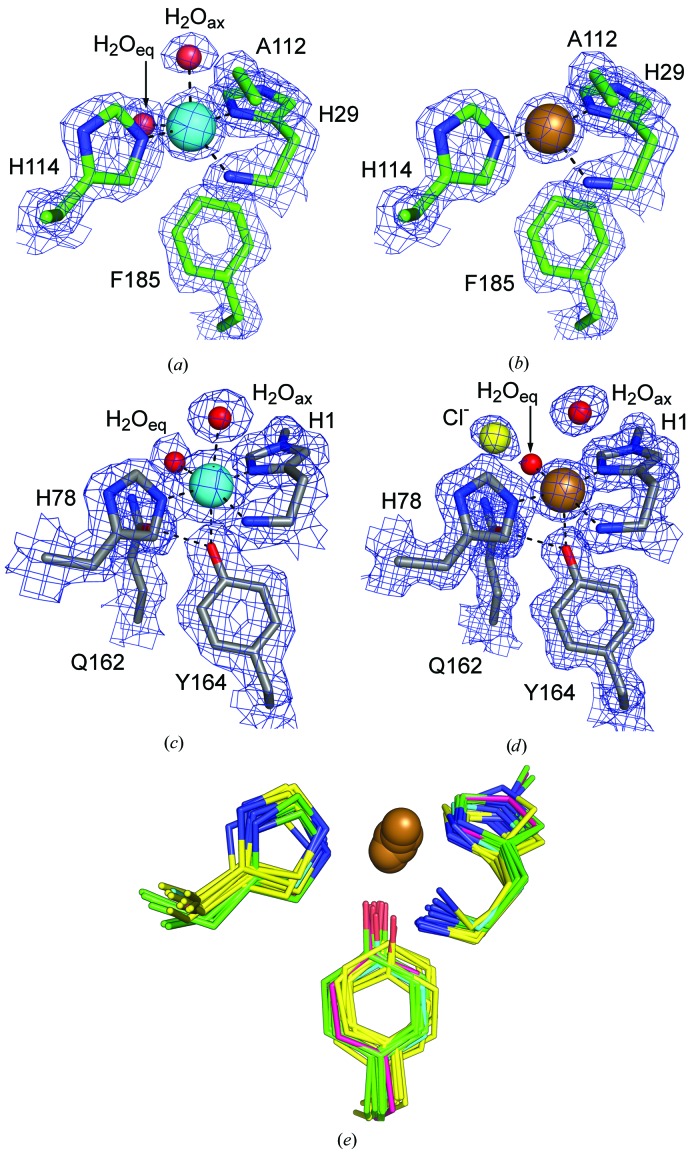
The copper-binding site in LPMOs. (*a*) The copper(II)-binding site (PDB entry 4alc) and (*b*) the photoreduced copper(I)-binding site (PDB entry 4alt) of *Ef*AA10_A. (*c*) The copper(II)-binding site (PDB entry 5acg) and (*d*) the partially photoreduced copper(II)/(I)-binding site (PDB entry 5ach) of *Ls*AA9_A. The copper spheres are in cyan to indicate copper(II) and in a copper colour to indicate copper(I). All electron-density maps (2*F*_o_ − *F*_c_) are contoured at the 1.5σ level. (*e*) Superposition of all structurally characterized copper-loaded LPMOs. AA9 members (PDB entries 4eir, 4qi8, 4eis, 4d7u, 4b5q, 3zud and 5ach) are shown in green, AA10 members (PDB entries 5fjq, 4alt, 5aa7, 4oy6, 4oy7, 5ftz, 4gbo and 4x27) in yellow, *Ao*AA11 (PDB entry 4mai) in cyan and *Ao*AA13 (PDB entry 4opb) in magenta. See Table 1 ▸ for the protein names of the AA9 and AA10 members.

As highlighted above, while the first structures from the AA9 family and the mutagenesis thereof led to the understanding that a divalent metal-binding site was crucial for activity, the nature of this metal was not clear. At the time when oxidative cleavage was demonstrated for AA10 there was still debate as to the nature of the active metal. In 2011 a number of publications on AA9 LPMOs firmly demonstrated that the active metal was copper using a variety of methods including metal identification in isolated native protein activity assays, binding studies and structural/spectroscopic studies (Westereng *et al.*, 2011 ▸; Phillips *et al.*, 2011 ▸; Quinlan *et al.*, 2011 ▸). Shortly afterwards, activity studies showed that copper was also the active metal in AA10 LPMOs (Vaaje-Kolstad *et al.*, 2012 ▸). The identification of copper as the active-site metal ion was corroborated by structure determination of the first copper-loaded LPMO, *Ta*AA9_A (Quinlan *et al.*, 2011 ▸), demonstrating that catalysis is mediated by a deceivingly simple-looking metal centre: a copper ion coordinated by a motif christened the ‘histidine brace’ (His brace hereafter; Fig. 3 ▸). The *K*_d_ for Cu^2+^ was estimated by ITC to be less than 1 n*M*, while at pH 5 no binding was observed with Mg^2+^, Ca^2+^, Mn^2+^, Co^2+^, Ni^2+^ or Zn^2+^.

Two structures of *Ta*AA9_A were determined: one of the protein as purified, in which a low-occupancy copper was modelled, and one in the presence of a high concentration of copper (Table 1 ▸), showing disorder which was modelled as copper in a main conformation and an alternative (lower occupancy) conformation. Copper sites are classified into several types (Crichton, 2012 ▸), and type 2 sites are mononuclear and are often coordinated by multiple histidines (three or four) in a square-planar or tetragonal geometry. Type 2 copper sites additionally have a very characteristic EPR signature. Apart from the number of His residues that are involved, the structure and EPR spectroscopy of *Ta*AA9_A were typical of a type 2 copper site with Jahn–Teller distorted octahedral geometry (elongation of the distance to the axial ligands) consistent with an at least partial copper(II) state, but with some disorder both of the copper and its exogenous ligands. Despite its limitations, this first structure of a Cu-loaded LPMO was very significant. For example, it was used to build active-site models for AA9, which could then be subjected to density functional theory calculations in order to investigate various aspects of the mechanism (Kim *et al.*, 2014 ▸; Kjaergaard *et al.*, 2014 ▸), in one case with additional experimental information derived from XANES and EXAFS in solution (Kjaergaard *et al.*, 2014 ▸).

The first studies of the metal centre of an AA10 enzyme were performed on *Sm*AA10_A. HSQC NMR spectra showed the binding of several metals at the His brace with decreasing *K*_d_ values for Ca^2+^ (greater than 10 m*M*) > Mg^2+^ > Fe^3+^ > Co^2+^ >> Zn^2+^ > Cu^2+^. The *K*_d_ values for Zn^2+^ and Cu^2+^ were determined by ITC as 330 and 55 n*M*, respectively. The *K*_d_ for Cu^+^ was estimated indirectly as 1.2 n*M* (Aachmann *et al.*, 2012 ▸). The article reporting the first X-ray structure of a copper-loaded AA10 from *Bacillus amyloliquefaciens* (Hemsworth, Taylor *et al.*, 2013 ▸) also measured the affinity for Cu^2+^ to be 6 n*M* at pH 5 by ITC, with Zn^2+^ being the only other tested divalent metal ion with measurable binding. Binding of metals was also indicated by an increase in *T*_m_ by 20 K for Cu^2+^ and 7 K for Ni^2+^ and Zn^2+^. There is thus a strong preference for copper, but some LPMOs are able to bind other ions (primarily zinc and nickel), in agreement with some of the ions bound in the early structures of AA9 members.

In *Ba*AA10_A the metal is photoreduced and shows a T-shaped coordination geometry in the structure, being coordinated solely by the His brace. EPR in solution showed a mononuclear copper(II) ion in a single binding site with a distorted axial coordination geometry with characteristics that were between type 1 and type 2, but closer to type 2 according to the authors. Since AA9 structures with geometry compatible with copper(II) were available at the time, Hemsworth, Taylor *et al.* (2013 ▸) suggested that photoreduction is easier for members of the AA10 family than the AA9 family, probably reflecting mechanistic differences.

Clearly, photoreduction of the active-site copper(II) to copper(I) is a challenge in LPMO structural research. While one can argue that the copper(I) photoreduced state is catalytically relevant, the catalytically relevant oxygen-bound species are predominantly expected to be copper(II) species (see below), and it is likely that many of the structures of LPMOs deposited in the PDB represent mixture of states to some extent, complicating structural interpretation. In Table 1 ▸ we give the presumed predominant oxidation state in all determined crystal structures of LPMOs, our criteria (in the footnote to Table 1 ▸) being somewhat stricter than those of Gudmundsson *et al.* (2014 ▸). Copper-loaded structures are available for all four families of LPMOs known to date, but copper(II) structures are only available for a few representatives and not at all for AA11 and AA13. A careful study was carried out for *Enterococcus faecalis* AA10_A, which not only succeeded in obtaining the first structure of an AA10 in a predominantly copper(II) state by reducing the dose and by helical data collection, but also elegantly showed the evolution of photoreduction with a series of six structures collected from the same crystal with increasing X-ray dose (Gudmundsson *et al.*, 2014 ▸). The structure of the copper(II) binding site is described as trigonal bipyramidal, although with significant distortion of the bonding angles (Fig. 3 ▸ *a*). As noted already in Hemsworth, Taylor *et al.* (2013 ▸), the exogenous ligands of the copper ion cannot have the same geometry in AA10 as in AA9 because of the steric constraints of a conserved Ala (shown also in Figs. 3 ▸ *a* and 3 ▸ *b*). The least and most photoreduced structures (Figs. 3 ▸ *a* and 3 ▸ *b*) of the active-site copper were subjected to quantum-mechanical calculations (Gudmundsson *et al.*, 2014 ▸) and resulted in charges for the copper ion of +1.48 and +0.99, respectively, for the copper(II) and copper(I) forms, which is in excellent agreement with the results obtained by similar methods for *Ta*AA9_A by Kim *et al.* (2014 ▸), where the derived charges on the copper(II) and copper(I) states are +1.48 and +0.92, respectively. Recently low X-ray dose structures showing predominantly copper(II) with very little disorder have also been reported for *Lentinus similis* AA9_A (*Ls*AA9_A), which is shown for reference (Figs. 3 ▸ *c* and 3 ▸ *d*; Frandsen *et al.*, 2016 ▸).

Structure determination of many LPMOs in AA9, AA10 and the newer families AA11 (chitin-acting) and AA13 (starch-acting) with bound copper have shown a remarkable conservation of the basic copper-binding motif regardless of specificity. The His brace forming the metal-binding site and its arrangement are extremely similar in all determined LPMO structures (Fig. 3 ▸ *e*). With regard to the aromatic residue at the metal-binding site, all AA9, AA11 and AA13 enzymes which have been structurally characterized have a Tyr residue, with the hydroxyl being at a borderline distance for coordination to copper. Most AA10 family members have, like *Sm*AA10_A (Vaaje-Kolstad, Houston *et al.*, 2005 ▸), a Phe instead of a Tyr (90% conservation), and an Ala preceding the second active-site His (Hemsworth, Taylor *et al.*, 2013 ▸), a combination which prevents an identical coordination geometry of exogenous ligands to that in AA9. In AA9 and AA13, a conserved Gln residue two residues before in the sequence hydrogen bonds to the active-site Tyr (shown in Figs. 3 ▸ *c* and 3 ▸ *d*), while in AA11 the corresponding Glu fulfils the same role, indicating the importance of the Tyr in the active site of these families. The corresponding residue to Gln varies both in identity and in conformation in AA10, which is indicative of a less strict functional role. AA9 structures additionally have a conserved His that hydrogen bonds to the Gln. Interestingly, *Sc*AA10_B, which is active on cellulose, and *Tf*AA10_A (also known as E7; PDB entry 4gbo; P. M. Alahuhta & V. V. Lunin, unpublished work) have a Tyr instead of a Phe and preserve the hydrogen-bonding network to the active-site Tyr (both Gln and His), as well as having a type 2 Cu EPR spectrum similar to that reported for *Ta*AA9_A (Forsberg, Mackenzie *et al.*, 2014 ▸). Just as mutagenesis of Tyr to Phe in *Tt*AA9_E impaired activity, mutation of Phe to Tyr in chitin-active AA10 enzymes impairs activity (Forsberg, Røhr *et al.*, 2014 ▸).

A very recent publication suggested that AA10 is heterogenous in its copper binding, even though only one active-site copper(II) conformation was observed by X-ray crystallo­graphy (Chaplin *et al.*, 2016 ▸). However, EPR spectra are best simulated with two similarly abundant solution species, one of which only coordinates to two side-chain N ligands (Chaplin *et al.*, 2016 ▸). To our knowledge, the only crystallographic observation of an LPMO copper coordinated by two ligands to date is the minor conformation in the disordered copper at the active site of *Ta*AA9_A, which is too distant (3.6 Å) from the N-terminus for coordination (Quinlan *et al.*, 2011 ▸).

Most structures of characterized fungal LPMOs show an unusual post-translational modification: methylation at N^∊1^ of the N-terminal histidine (see, for example, Quinlan *et al.*, 2011 ▸; Hemsworth *et al.*, 2014 ▸; Lo Leggio *et al.*, 2015 ▸). The role of this modification is currently unclear, but at least three AA9 members which have been expressed in *Pichia pastoris* and one AA11 expressed in *Escherichia coli* do not have this modification, and show activity nonetheless (Bennati-Granier *et al.*, 2015 ▸; Westereng *et al.*, 2011 ▸; Borisova *et al.*, 2015 ▸; Hemsworth *et al.*, 2014 ▸).

## 3. Substrate binding and catalysis   

### 3.1. Initial identification of a substrate-binding surface   

In order to fully understand and describe the mode of action of LPMO enzymes, thorough characterization of their protein–substrate interactions and specificity are needed. As described above, the first structure of an AA10, *Sm*AA10_A, revealed a conserved patch of hydrophilic residues that were proven to be involved in substrate binding by mutagenesis (Vaaje-Kolstad, Houston *et al.*, 2005 ▸). The *Sm*AA10_A–chitin interaction at this surface was later mapped by NMR spectroscopy by monitoring deuterium exchange after binding of β-chitin, providing direct experimental evidence for binding at this surface for the first time (Aachmann *et al.*, 2012 ▸). A mutagenesis study involving *Tt*AA9_E clearly showed the importance of an aromatic residue in the CBM1-like motif extending from the corresponding putative substrate-binding surface (Harris *et al.*, 2010 ▸), and the distribution of aromatics has been discussed in detail (for example, in Li *et al.*, 2012 ▸). For AA11 the active-site surface is slightly more convex and is devoid of aromatic residues, but has a number of polar residues that are potentially able to make polar interactions with the substrate similarly to AA10 (Hemsworth *et al.*, 2014 ▸). A consensus now exists that many LPMOs interact with their crystalline substrates at relatively flat surfaces, and that binding takes place either through stacking interactions with aromatic residues (*e.g.* AA9) and/or by polar interactions with hydrophilic residues (*e.g.* AA10 and AA11).

### 3.2. Substrate specificity: cellulose, starch and chitin   

AA9 was discovered as a family of cellulose-degrading LPMOs, while AA10 was discovered as a chitin-degrading family, although shortly after the discovery of the oxidative degradation of chitin by *Sm*AA10_A the Eijsink group also showed that other AA10 members could degrade cellulose (Forsberg *et al.*, 2011 ▸). As can be seen in the overview of specificities in Table 1 ▸, it still holds that most AA9 family members are cellulose-degrading and AA10 family members degrade chitin or cellulose.

**Figure 4:**
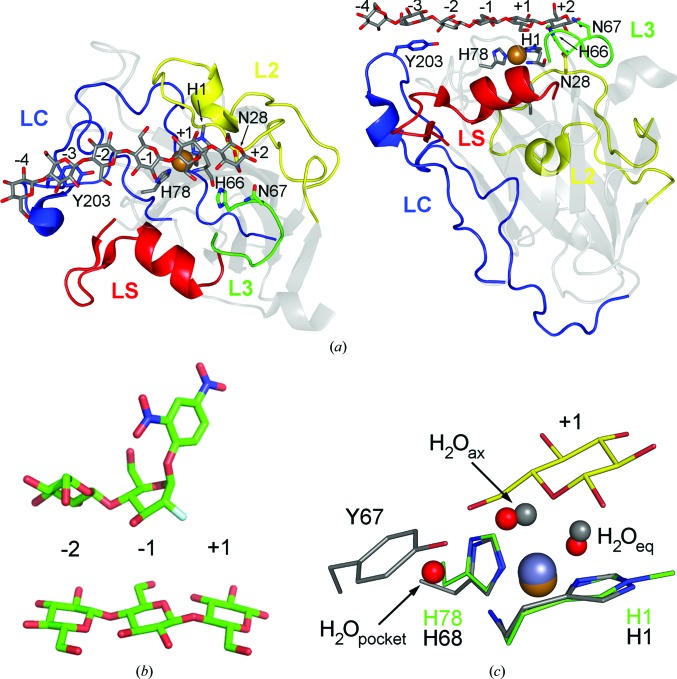
Substrate binding by *Ls*AA9_A. (*a*) Top and side view of G6 binding in *Ls*AA9_A (PDB entry 5aci): *Ls*AA9_A is shown in grey with the loops L2, L3, LS and LC coloured yellow, green, red and blue, respectively. The cellohexaose substrate, the His brace and selected substrate-interacting residues are shown as sticks. Residues are coloured in accordance with the structural elements to which they belong. (*b*) Comparison of glycoside units binding at the −2/+1 subsite in *Ls*AA9_A (bottom; PDB entry 5acf) and the Michaelis–Menten complex of endoglucanase Cel5A from *Bacillus agaradhaerens* with 2′,4′-dinitrophenyl-2-deoxy-2-fluoro-β-d-cellobioside (PDB entry 4a3h; Davies *et al.*, 1998 ▸). (*c*) Comparison of the active sites of *Ls*AA9_A (with protein in green and waters in red; PDB entry 5acg) and *Tt*AA9_E (PDB entry 3eii; chain *B*; all in grey). The glucosyl unit in subsite +1 of *Ls*AA9_A–copper(II)–G3 (PDB entry 5acf) is superimposed and shown in yellow.

Shortly after the observation of the CBM1-like tyrosines in *Tt*AA9_E (Harris *et al.*, 2010 ▸), the structure of *Ta*AA9_A (Quinlan *et al.*, 2011 ▸) revealed another tyrosine-containing loop on the same surface but on the opposite side with respect to the active site. An equivalent loop and tyrosine were also found in *Nc*AA9_M in a structural study of AA9s from *Neurospora crassa* (*Nc*AA9_D and *Nc*AA9_M). Here, the loop was denoted L2 (this and other loop positions are marked in Fig. 4 ▸ for *Ls*AA9_A), and it was suggested that the aromatic residues were spatially positioned to accommodate stacking inter­actions with glucose units within the crystalline cellulose (Li *et al.*, 2012 ▸). In the same publication it was further suggested that an insertion in the cellulose-active *Sc*AA10_C compared with *Sm*AA10_A (extending from a region equivalent to loop L2 in AA9) could account for cellulose specificity. Book *et al.* (2014 ▸) similarly suggested that this insertion in AA10 members accounted for cellulose specificity and classified this region as motif 1. The Sandgren group showed from MD simulations based on the *Pc*AA9_D structure that the loops L2, LS and LC (the latter harbouring a tyrosine residue which is conserved in most of the structurally characterized AA9 enzymes) had essential roles in interacting with crystalline cellulose (Wu *et al.*, 2013 ▸).

Comparative studies have since been carried out on AA10s active on chitin (*Sm*AA10_A and *Bl*AA10_A) and on cellulose (*Sc*AA10_C and *Tf*AA10_B) (Forsberg, Røhr *et al.*, 2014 ▸). The EPR spectra of the cellulose-active AA10s described were similar to those of the cellulose-active *Ta*AA9_A and distinct from those of chitin-active AA10s. At the same time, it was found that *Sc*AA10_C was able to bind chitin in a nonproductive manner. Based on this, it was proposed that specificity is not defined by the ability of the enzymes to bind substrates, but rather that the copper-centre configuration is a determinant of substrate specificity. As the residues directly involved in copper binding appeared to be identical, it was speculated that positions more remote from the copper were indirectly affecting the active site, causing the differences in substrate specificity. The structures of two cellulose-active AA10s (*Sc*AA10_B and *Sc*AA10_C) were published in the same year (Forsberg, Mackenzie *et al.*, 2014 ▸). Despite the EPR spectra of the two enzymes being similar, the active sites showed clear structural differences, with the active site of *Sc*AA10_C being similar to that of *Sm*AA10_A, while *Sc*AA10_B resembled AA9. Structural comparisons revealed a cavity in the chitin-active AA10 (not found in the cellulose-active AA10s) near the active site, which was proposed to accommodate the *N*-acetyl group of the substrate (Forsberg, Mackenzie *et al.*, 2014 ▸), but was later shown not to be present in the chitin-active *Cj*AA10_A (Forsberg *et al.*, 2016 ▸). Forsberg, Mackenzie *et al.* (2014 ▸) also noted that the cellulose-active *Sc*AA10_C had an insertion between strands β6 and β7 (relative to *Sm*AA10_A), positioned spatially equivalent to the LS loop in AA9s, and proposed that substrate specificity was not correlated with the copper centre, but depended on substitutions more remote from the active site affecting substrate interaction. Interestingly, Forsberg *et al.* (2016 ▸) found that *Cj*AA10_A, as well as lacking the proposed chitin-binding cavity, had an extended flat substrate surface with features of both cellulose-active and chitin-active AA10s, but was only active on chitin. They further made the interesting observation that the catalytic centres of *Cj*AA10_A and AA10s of viral origin are remarkably similar.

With respect to AA11, it is interesting to note that the EPR spectrum of *Ao*AA11 groups together with those of cellulose-active LPMOs (Forsberg, Mackenzie *et al.*, 2014 ▸). Also, considering the L2-equivalent loop (Hemsworth *et al.*, 2014 ▸), that in *Ao*AA11 appears to resemble that of *Sm*AA10_A more than that of *Sc*AA10_C, which is consistent with the experimentally measured chitinolytic activity.

**Figure 5:**
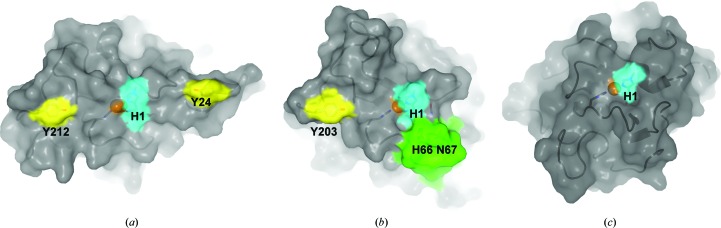
Features of the substrate-binding surfaces of LPMOs. (*a*) Surface of *Ta*AA9_A (PDB entry 3zud) with tyrosines at the substrate-binding surface coloured yellow. (*b*) Surface of *Ls*AA9_A (PDB entry 5aci) with Tyr203 in yellow and the L3 loop in green, with His66 and Asn67 in stick representation. (*c*) Surface of *Ao*AA13 with a groove spanning the active site. In all cases the N-terminal histidine (His1) of the histidine brace is shown in stick representation and coloured cyan and the coppers are shown as spheres.

The initial paradigm for LPMOs was that their function is to attack crystalline substrates and favour access by glycoside hydrolases, and as such they possess flat binding sites. Generally speaking, it is said that β-1,4-linked substrates such as cellulose and chitin have a higher tendency to form crystalline structures and thus are harder to access than most starches, which are α-1,4-linked (with additional α-1,6 linkages) and often more digestible, although more recalcitrant forms of starch exist (Pérez & Bertoft, 2010 ▸; Vu & Marletta, 2016 ▸). Starch-active LPMOs (AA13) were first reported in the academic literature by Vu, Beeson, Phillips *et al.* (2014 ▸). The first (and so far only) available structure of an AA13 is that from *Aspergillus oryzae* (*Ao*AA13) and was reported by Lo Leggio *et al.* (2015 ▸). In the *Ao*AA13 structure, no obvious aromatic residues were present at the putative substrate surface. However, this surface appears to be more contoured in AA13s than in other LPMOs that do not act on α-1,4 linkages. In fact, a shallow groove spanning the active site in *Ao*AA13 is likely to play a role in substrate interaction of starch substrates (Fig. 5 ▸), although to date there is no experimental evidence. The groove has a size that fits a single amylose chain, although an amylose double helix has also been proposed to bind (Vu & Marletta, 2016 ▸). Understanding of the AA13 family is lagging behind, but hopefully again the structural studies will guide further biochemical and mutagenesis studies and help us to understand function.

### 3.3. Regiospecificity   

Up to now, we have barely touched on the subject of regiospecificity, namely the preference of LPMOs to oxidize at C1 or C4 or both. In Table 1 ▸, the reported experimental regiospecificity for all structurally characterized LPMOs is given. The first AA10 enzymes to be characterized, for example the chitin-active *Sm*AA10_A (Vaaje-Kolstad *et al.*, 2010 ▸) and the cellulose-active *Sc*AA10_C (Forsberg *et al.*, 2011 ▸), seemed to exclusively oxidize at C1. In contrast, by 2012 AA9s were known to oxidize at C1, C4 or C1/C4, leading to the suggested sequence-based subfamily classification as types 1, 2 or 3, respectively, for the three oxidation modes (Phillips *et al.*, 2011 ▸; Li *et al.*, 2012 ▸; Vu, Beeson, Phillips *et al.*, 2014 ▸). The structures revealed conserved structural features correlating with AA9 C1/C4 regiospecificity such as loop L2 in type 3 LPMOs (Vu, Beeson, Phillips *et al.*, 2014 ▸).

Later, three modular AA9 proteins (AA9-CBM1) from *Podospora anserina* active on cellulose were characterized (Bennati-Granier *et al.*, 2015 ▸). Of the three proteins, *Pa*AA9_E released C1-oxidized products, while *Pa*AA9_A and *Pa*AA9_H both released C1- and C4-oxidized products. While the sequence and regiospecificity of *Pa*AA9_A and *Pa*AA9_E are in agreement with the previous classification (Vu, Beeson, Phillips *et al.*, 2014 ▸), *Pa*AA9_H was classified on the basis of sequence as a type 2 AA9, which predicts C4 oxidation only, showing that sequence alone may not be sufficient to predict the regiospecificity.

AA10 members generally appear to oxidize mostly at C1, although a double oxidizing ability has been found for some members. To our knowledge, no member of AA10 has been reported to oxidize at C4 alone. The first demonstration of C4 oxidation for an AA10 member was for *Sc*AA10_B, which oxidizes cellulose at C1/C4 (Forsberg, Røhr *et al.*, 2014 ▸). It was also shown that the C1-oxidizing *Sc*AA10_C and the C1/C4-oxidizing *Sc*AA10_B act in synergy on cellulose (PASC), indicating that these enzymes recognize different regions of the substrates (Forsberg, Røhr *et al.*, 2014 ▸). The synergistic effect was correlated to structural variation of the copper active-site surroundings. Of special interest was a conserved alanine in AA10s that was proposed to limit access to the axial position on the copper (Hemsworth, Davies *et al.*, 2013 ▸; Forsberg, Mackenzie *et al.*, 2014 ▸; Forsberg, Røhr *et al.*, 2014 ▸), although it still allows the copper of C1-oxidizing AA10 to bind two water molecules (Gudmundsson *et al.*, 2014 ▸). Structural comparison showed that this alanine was displaced (∼2.5 Å for C^β^ in *Sc*AA10_C_relative to *Sc*AA10_B) owing to the neighbouring residues adopting a different conformation. From the structural observations, it was postulated that the ability of copper to bind a ligand in the axial position could be a determinant of C4-oxidizing activity, and that the degree of accessibility to the axial position on the copper determines the regiospecificity of AA10s, simultaneously suggesting that a similar correlation would exist for other families (*e.g.* for AA9).

When the structure of *Nc*AA9_C was determined (Borisova *et al.*, 2015 ▸), a correlation was indeed found. The authors observed that an Ala or Asp at a position packing against the internal active-site His (as in *Nc*AA9_C and *Nc*AA9_D, respectively) would allow an axial ligand, leading to C4 oxidation, and a partially open axial position (with Pro at this position) would lead to C1/C4 oxidation (as in *Ta*AA9_A), while a Tyr would block the axial position, leading to C1 oxidation (as in *Pc*AA9_D or *Tt*AA9_E). However, it must be noted that for *Tt*AA9_E a slightly distorted axial coordination to the metal (zinc in this case) is possible (see Fig. 4 ▸ *c*), and in *Nc*AA9_F (a likely C1 oxidizer), which was not included in the Borisova analysis since this structure was published almost at the same time, the axial water is present and in fact interacts with the corresponding Tyr. Thus, the coordination is likely to be affected but not blocked as such. The few characterized members of AA11 and AA13 release C1-oxidized products. No determinants of regiospecificity have yet been proposed, given the lack of experimental evidence.

### 3.4. Soluble substrates   

Initially, several LPMOs had been characterized as acting on insoluble substrates. *Nc*AA9_C was then reported to act on both cellulose and small soluble cellooligosaccharides (Isaksen *et al.*, 2014 ▸). Agger and coworkers later reported *Nc*AA9_C activity on (1→3, 1→4)-β-d-glucan (MLG) and on certain hemicelluloses such as xyloglucans (XG) and to lesser extent glucomannan (Agger *et al.*, 2014 ▸). Similar to *Nc*AA9_C, *Pa*AA9_H (Bennati-Granier *et al.*, 2015 ▸) also showed activity on soluble substrates such as cellooligosaccharides [degree of polymerization (DP) of 4–6] and certain hemicelluloses such as XG, glucomannan, MLG and lichenan. Unfortunately, no structure of *Pa*AA9_H is available.

The structure of *Nc*AA9_C was published by Borisova *et al.* (2015 ▸), and showed that an insertion, denoted loop L3 (which is absent in AA9s that do not act on soluble substrates, for example *Ta*AA9_A), was involved in forming the substrate-binding surface. Although interaction with substrate was measured with micromolar affinity, no complex structures were obtained. We finally managed to determine a crystallo­graphic complex with an AA9 LPMO from *Lentinus similis* (*Ls*AA9_A), the first of the kind (Frandsen *et al.*, 2016 ▸). *Ls*AA9_A is also active on cellulose and soluble cellooligosaccharides (>DP2), and the structures revealed several polar residues around the active site interacting with cellotriose (G3) and cellohexaose (G6) at subsites −1 to +2 and −4 to +2, respectively. In this notation, cleavage occurs between subsite −1 and subsite +1, with ‘−’ corresponding to the nonreducing end and ‘+’ corresponding to the reducing end (Davies *et al.*, 1997 ▸), in analogy with GHs. The *Ls*AA9_A–G6 structure revealed that the glucosyl unit at subsite −3 was stacking with the surface-exposed tyrosine (in the LC loop of most AA9s), confirming the involvement of this aromatic residue in substrate interaction. Intriguingly, however, this residue is missing in the cellulose-active *Nc*AA9_F (Tan *et al.*, 2015 ▸), even though it is conserved in all other determined AA9 structures. In addition, in LsAA9_A complexes, the glucosyl unit at subsite +1 stacked directly on top of the methylated His1 (O5 lone pair–aromatic interaction), while several polar residues made hydrogen bonds to the substrate at the rest of the subsites. The terminal glucosyl unit at the reducing end of the substrates was anchored at subsite +2 through hydrogen bonds to Asn28, His66 and Asn67 (Fig. 4 ▸ *a*).

Both the chair conformations and the glycosidic torsion angles of the complexes in Frandsen *et al.* (2016 ▸) very closely resemble ideal values, showing that complex formation drives very little distortion of the substrate. This is highly exceptional in enzyme catalysis, as a comparison with the Michaelis–Menten complex of a classic glycoside hydrolase easily illustrates (Fig. 4 ▸ *b*). Thus, the energy for breaking the glycosidic linkage must fully come from the exceptional chemistry of the copper–oxygen activation. With the first structure of an LPMO–carbohydrate complex structure at almost atomic level resolution combined with spectroscopic methods (EPR), substantial and detailed insights into the mechanism of action of LPMOs were obtained (Frandsen *et al.*, 2016 ▸), which are further elaborated in the next section.

The *Ls*AA9_A complexes also confirmed the involvement of loop L3 in substrate binding, as had been speculated for *Nc*AA9_C (Borisova *et al.*, 2015 ▸), as this loop formed a structural ridge interacting with the glucosyl unit at subsite +2 (Figs. 4 ▸ and 5 ▸), which in fact is also present in *Nc*AA9_D (Li *et al.*, 2012 ▸). It would be interesting to determine whether *Nc*AA9_D might also have activity on cellooligosaccharides, which to our knowledge has not been reported. Shortly after the publication of the *Ls*AA9_A complexes, an NMR study on *Nc*AA9_C showed relatively similar interactions with cellohexaose (G6) and xyloglucans (XG14, polyXG), although G6 did not span as far as the conserved surface Tyr (rather, the data suggested binding from −3 to +3; Courtade *et al.*, 2016 ▸). Interestingly, Courtade and coworkers also showed significant chemical shift differences for certain residues in the L3 loop. In Isaksen *et al.* (2014 ▸) it was suggested that three conserved Asn residues in *Nc*AA9_C are involved in the binding of cellooligosaccharides. In Courtade *et al.* (2016 ▸) these residues were not reported to be affected by NMR titration. In *Ls*AA9_A only the central one of these equivalent Asn residues (Asn28) is involved in the binding of G6/G3, while in *Pa*AA9_H they are substituted by Ser25, Asn26 and Phe27, indicating that only the central Asn is involved in the binding of cellooligosaccharides. Lacking structural data, Bennati-Granier *et al.* (2015 ▸) speculated that loop L3 (in *Pa*AA9H spanning Gly64–Ser83) with the polar residues Glu66, Asp75 and Asp77 (the equivalent residues in *Nc*AA9_C are Glu65, Asp74 and Asp76) was responsible for XG specificity. The interaction of *Ls*AA9_A Asn67 with substrate (equivalent to *Nc*AA9_C Glu65) demonstrates that this residue contributes to the specificity towards substrates with a β-(1,4)-linked glucose backbone rather than solely XG. From structural comparison with *Ls*AA9_A, it would seem that the aspartates could be involved in XG specificity (possibly interacting with substitutions originating from subsites −2 or +3).

### 3.5. Electron donors/redox partners   

In order to catalyse the oxidation of polysaccharides, LPMOs are dependent on redox partners that donate electrons which are used to reduce the active-site copper and to activate molecular oxygen. These electron donors range from small molecules (ascorbate, gallate, reduced glutathione and others) to insoluble lignin polymers and endogenous modular macromolecules (Vaaje-Kolstad *et al.*, 2010 ▸; Phillips *et al.*, 2011 ▸; Langston *et al.*, 2011 ▸; Quinlan *et al.*, 2011 ▸; Dimarogona *et al.*, 2012 ▸). When oxidative activity was first found for *Sm*AA10_A small-molecule electron donors were used, as is frequently performed when testing for LPMO activity. After lignin was identified to function as an electron donor, it was also shown that long-range electron transfer (ET) from lignin to LPMOs can occur (Westereng *et al.*, 2015 ▸). The first evidence for an endogenous redox partner came in 2011, when cellobiose dehydrogenase (CDH) was indicated by *in vivo* experiments to function as source of electrons for AA9s in *T. terrestris* and *N. crassa*, a function which is now well established. Li *et al.* (2012 ▸) first proposed putative electron pathways based on a conserved patch mapped on the structures, where the haem-containing domain of CDH was docked computationally. At least two potential CDH sites and pathways are reviewed in Beeson *et al.* (2015 ▸). In AA13 a putative electron-transfer pathway (Tyr224, Trp215, Trp83, Phe95 and Phe161) has also been proposed for *Ao*AA13 (Lo Leggio *et al.*, 2015 ▸). Tan *et al.* (2015 ▸) suggested direct electron transfer to the LPMO-active site from the haem-containing domain of CDH, and recent NMR spectroscopy studies also showed that CDH appears to interact with *Nc*AA9_C on the flat substrate surface (Courtade *et al.*, 2016 ▸). This finding is intriguing since this would imply competition of CDH and substrate or that all electrons are delivered before the substrate. Recent studies show that light-excited photosynthetic pigments are excellent electron donors and can considerably speed up the LPMO reaction (Cannella *et al.*, 2016 ▸), and a pathway for electron transfer involving His87 of *Ta*AA9_A or a similarly placed residue in other AA9 LPMOs was suggested. Intriguingly, LPMOs are extremely promiscuous when it comes to accepting electrons from CDHs; for example, *Nc*AA13 was able to accept electrons from *Myceliophthora thermophila* CDH-2 (Vu, Beeson, Phillips *et al.*, 2014 ▸). Recently, it has been shown that other dehydrogenases than CDH can function as redox partners for LPMOs (Kracher *et al.*, 2016 ▸; Garajova *et al.*, 2016 ▸).

### 3.6. Catalytic mechanisms   

The first elucidation of the enzymatic mechanism of LPMOs was achieved in 2010 (Vaaje-Kolstad *et al.*, 2010 ▸), when it was shown using isotope-labelled ^18^O_2_ that *Sm*AA10_A incorporates one O atom into the substrate (chitin), establishing LPMOs as monooxygenases. Although the exact catalytic mechanisms of LPMOs are unknown, proposals have been put forward suggesting that LPMOs oxygenate their substrates using activated oxygen species in a putative mechanism involving two electrons. Other naturally occurring, well characterized monooxygenases which accomplish two-electron oxidations of their substrate with mononuclear type 2 copper centres are amine oxidase (AmO; Shepard & Dooley, 2015 ▸), galactose oxidase (GO; Solomon *et al.*, 2001 ▸), peptidylglycine α-hydroxylating monooxygenase (PHM; Solomon *et al.*, 2014 ▸) and dopamine β-monooxygenase (DβM; Klinman, 2006 ▸). In AmO and GO a protein-derived cofactor functioning as a redox-active functional group is formed (in GO a covalent thioether bond is formed between a tyrosine and a cysteine) in an event known as cofactor biogenesis (Shepard & Dooley, 2015 ▸; Solomon *et al.*, 2014 ▸), allowing the enzymes to stabilize radicals forming during catalysis. PHM and DβM contain two mononuclear sites and are also known as noncoupled bi­nuclear copper enzymes. In these enzymes hydroxylation occurs by the first site and another electron is provided using long-range (∼11 Å in PHM) ET from the other site (Solomon *et al.*, 2001 ▸, 2014 ▸; Chen & Solomon, 2004 ▸). In LPMOs no additional redox centres or intramolecular sites for ET have been reported. The ability of LPMOs to catalyse reactions despite lacking the functionalities of other mononuclear monooxygenases (cofactor biogenesis or intramolecular ET sites), combined with their atypical ligand (bidentate coordinated N-terminal histidine), makes them unique in terms of their copper chemistry and explains the attention that they have gained in the field of bioinorganic chemistry. The first LPMO mechanism proposed involved a copper(II)-superoxo species abstracting an H atom from the substrate followed by hydroxylation of either C1 or C4 (Phillips *et al.*, 2011 ▸; Beeson *et al.*, 2012 ▸). In support of this mechanism, in the structures of AA9s from *N. crassa* (*Nc*AA9_C and *Nc*AA9_M) dioxygen species (superoxide and peroxide) were modelled in elongated electron density by the axial position on the copper (Li *et al.*, 2012 ▸). Later, Kjaergaard *et al.* (2014 ▸) showed using a spectroscopic and computational study that the unique bidentate N-terminal ligand leads to a T-shaped copper(I) site and is advantageous in strong oxygen binding with minimal reorganization energy. Concurrently, an oxygen-rebound mechanism involving a copper(II)-oxyl species [in equilibrium with copper(III)-OH] was shown from calculations to be energetically more favourable (Kim *et al.*, 2014 ▸). A copper(III) species, although so far mostly proposed for small-molecule model complexes (Donoghue *et al.*, 2011 ▸; Dhar & Tolman, 2015 ▸), has been hypothesized for DβM (Kamachi *et al.*, 2005 ▸; Yoshizawa *et al.*, 2006 ▸; Itoh, 2006 ▸) and also speculated for LPMOs in Quinlan *et al.* (2011 ▸). Recently, it was suggested that the copper(II)-oxyl ↔ copper(III)-OH tautomerization described by Dhar & Tolman (2015 ▸) and Gagnon & Tolman (2015 ▸) could take place *via* proton abstraction from the LPMO amino-terminus [H_2_N-copper(II)-O ↔ HN-copper(III)-OH]. Interestingly, from a structural perspective, the *Ls*AA9_A–copper(II)–G3 (PDB entry 5acf) structure shows a hydrogen-bonding network linking the substrate to the amino-terminus (through a water molecule denoted as the ‘pocket water’), thus supporting this notion. The *Ls*AA9_A–copper(II)–G3 structure in Frandsen *et al.* (2016 ▸) showed that the binding of a glucosyl unit at subsite +1 placed the C6 close to copper, displacing the axial ligand (Fig. 4 ▸) and inducing a shortening of the Cu–Tyr distance. At the same time, the binding of a heavier chloride ligand at the equatorial position, which could be taken to mimic superoxide, was observed. This species was also clearly visualized by EPR spectroscopy, providing insights into the mode of action of LPMOs. This implies that the ability to coordinate/displace a water molecule in the axial position would be a prerequisite for function and is evidence against the binding of molecular dioxygen in the axial position, as proposed by Li *et al.* (2012 ▸). Displacement of the axial water when binding the substrate may occur regardless of regio­specifity, although experimental evidence for C1 oxidizers is lacking. In the C1 oxidizers *Tt*AA9_E and *Nc*AA9_F axial ligands to the copper are visible in some of the structures, although limitation of axial access has been proposed as a determinant of regiospecificity (Borisova *et al.*, 2015 ▸). The hydroxyl group of the tyrosine side chains proposed to block the axial water access could instead have a similar function to the ‘pocket water’ in *Ls*AA9_A (see Fig. 4 ▸ *c*). In this respect, it is also interesting to note that in *Ao*AA13 the backbone carbonyl of a glycine is also spatially positioned similarly to the ‘pocket water’ of *Ls*AA9_A.

In conclusion, the exact mechanism and whether it is exactly preserved in all LPMOs is still uncertain, and several routes regarding the formation of the oxygen species have been proposed and recently reviewed (Beeson *et al.*, 2015 ▸; Walton & Davies, 2016 ▸).

## 4. A final survey of available structures with a special focus on the last two years   

Table 1 ▸ collects information on all LPMO structures determined to date, most of which have already been discussed in some detail in previous sections of this review. As well as their known specificity, the table attempts to collect information of interest to a crystallographer, including the active-site metal modelled and its occupancy, the resolution and the crystallization conditions. Structures are available for 25 individual LPMO family members, including nine AA9s, 14 AA10s, one AA11 and one AA13, with a total of 56 PDB entries. Of the nine individual AA9 members, seven have a copper-loaded structure, of which three are mainly in a copper(II) state, while of the 14 AA10 members nine are available as copper-loaded and two as copper(II), somewhat supporting the suggestion in Hemsworth, Taylor *et al.* (2013 ▸) that AA10s are easier to photoreduce. The single AA11 and AA13 LPMOs for which a structure is available have a photoreduced copper(I).

The highest resolution record goes to the 0.95 Å resolution structure of *Ef*AA10_A, which is unfortunately devoid of metal in the active site (Vaaje-Kolstad *et al.*, 2012 ▸). The next highest resolution is for *Nc*AA9_D (Li *et al.*, 2012 ▸) and *Nc*AA9_F (Tan *et al.*, 2015 ▸), both of which were determined at 1.10 Å resolution and with copper. It is remarkable, and almost certainly a consequence of the compactness of the structure, that only four of the deposited structures have a resolution worse than 2.00 Å and none have a resolution worse than 2.50 Å. This count includes the structures of fusolin, a spindle-forming virulence factor found in insect viruses, which deserves a special mention in a review aimed at crystallographers, because fusolin is in a crystalline state in its native form and because of the technical achievement in determining the structure from natural crystals (Chiu *et al.*, 2015 ▸). The spindles were harvested from infected insects and larvae and purified by centrifugation with a sucrose gradient. Tiny crystals (typically ∼3 µm in diameter) were subjected to synchrotron diffraction with a microbeam (5–20 µm) after mounting on micromeshes and data were merged from multiple crystals. Apart from the challenges of data collection, the structure was then solved by *ab initio* molecular replacement in *PHENIX* (*Rosetta_MR*; Terwilliger *et al.*, 2012 ▸) with the structure of *Sm*AA10_A as a template, with which it shares only 14% sequence identity. Mature fusolin has a His brace and metal-binding site typical of the AA10 family, but uniquely among LPMOs forms a domain-swapped dimer through a C-terminal extension (Fig. 2 ▸ *d*). The structure presents a ‘typical’ flat LPMO surface with both polar residues and notably also Trp residues which could participate in chitin binding. However, bidentate coordination from the carboxylic side chain of a Glu from a symmetry-related molecule replaces the waters commonly found as ligands in this position in nonphotoreduced AA10s, and the glutamate side chain occludes the active site. Normally this would be regarded as an ‘accident’ of crystallization, but as this is a natural form of the protein the authors suggested that this is in fact a way in which fusolin is regulated; the LPMO is inactive in the crystalline spindle, but as it is released the active site becomes accessible and it can promote the degradation of chitin-rich matrices and thus promote infectivity. This hypothesis is supported by the fact that mutants of fusolin where the His brace is disrupted lose their biological function, but to our knowledge LPMO activity has not yet been demonstrated.

Very recently, the structure of the smallest LPMO domain structurally characterized to date, that of *Jonesia denitrificans* AA10_A, was determined (Mekasha *et al.*, 2016 ▸). This LPMO domain is part of a modular natural protein, but the domain in isolation, which is only 15.5 kDa in mass (142 amino-acid residues), is capable of C1 oxidation of both α-chitin and β-chitin. In Figs. 2 ▸(*a*) and 2 ▸(*b*), the structures of *Sm*AA10_A and *Jd*AA10_A are shown side by side to highlight the structural elements that are dispensable for substrate binding and catalysis. It is remarkable that such a small polypeptide is able to catalyze the oxidation of a glycosidic bond, and as such it presently represents the minimal structural requirements for a functional LPMO.

## 5. Perspectives, challenges and final remarks   

One question that will strike most structural biologists is why, if the His-brace motif is so simple, is it restricted to the same three-dimensional architecture? In other words, it seems reasonable to expect that we will eventually find LPMOs which are not structurally related overall to known LPMOs, but have the His brace and a similar mechanism. Structural motifs similar to the His brace have already been noted (Phillips *et al.*, 2011 ▸), in particulate methane monooxygenase (Smith *et al.*, 2011 ▸) and the bacterial copper resistance protein CopC (Zhang *et al.*, 2006 ▸). In either case, the relationship to the LPMO mechanism is not fully understood.

This review focuses primarily on the catalytic domains of LPMOs, but LPMOs are frequently modular enzymes with an AA domain at the N-terminus (this is important, since the N-terminus is one of the Cu ligands) and additional domains, which are often CBMs. AA9 LPMOs are associated with the typically cellulose-binding CBM1 in about a third of occurrences (Book *et al.*, 2014 ▸; Lo Leggio *et al.*, 2012 ▸). AA10 enzymes are also often associated with cellulose-binding CBM2 or CBM3 or chitin-binding CBM5 or CBM12, cumulatively in about a third of instances (Book *et al.*, 2014 ▸). AA13 enzymes owe their identification largely to the association with CBM20, a typical starch-binding CBM (Lo Leggio *et al.*, 2015 ▸; Vu, Beeson, Phillips *et al.*, 2014 ▸). Recently, the characterization of a module of unknown function associated with an LPMO from *C. japonicus* defined a new family of chitin-binding CBMs, CBM73 (Forsberg *et al.*, 2016 ▸). Removal of the CBM5 and CBM73 chitin-binding modules from full-length *Cj*AA10_A caused reduced LPMO activity on α-chitin (Forsberg *et al.*, 2016 ▸). The importance of CBMs for LPMO function was also underlined by a very recent study in which CBMs were deleted, appended or substituted in LPMOs (Crouch *et al.*, 2016 ▸).

With the determination of the first crystalline complex (Frandsen *et al.*, 2016 ▸), and of course building on a large body of biochemical, spectroscopic and structural knowledge contributed by many groups, the initial steps in the mechanism concurrent with and just after binding have been now de­lineated in detail, for one member of the LPMO family at least! However, a lot of work remains to perform in characterizing the next stages of the reaction to understand exactly the basis for substrate specificity, the oxygen activation by the His brace, the mechanisms of electron delivery and the release of products. In particular the α-1,4-glucan-active AA13 family remains extremely enigmatic: very little is known other than the products generated by a couple of enzymes and a single enzyme structure (Lo Leggio *et al.*, 2015 ▸) which is too different from the AA9 family to easily extend the recently obtained substrate interaction results to it.

Despite the fact that there are many high-resolution structures, there is no ultrahigh-resolution structure of a catalytically competent LMPO which could be used to unambiguously identify H atoms, yet to investigate the catalytic chemistry in detail small-molecule accuracy would be highly desirable. Photoreduction is likely to be problematic, owing to the high doses that will necessarily be involved, so a serial crystallo­graphy approach may be beneficial (Stellato *et al.*, 2014 ▸). Recently, good-resolution (2.1 Å) neutron diffraction data have been collected from a *Jd*AA10_A enzyme crystal (Bacik *et al.*, 2015 ▸), which may provide the first high-quality, room-temperature structure of a completely nonphotoreduced LPMO and additional information on hydrogen positions. Full elucidation of the catalytic mechanism and specificity in detail will necessitate further crystallographic studies with later reaction intermediates and soluble ligands. However, since many LPMOs naturally attack insoluble substrates, a full picture will be only obtained by the additional use of other techniques, such as NMR spectroscopy (Aachmann *et al.*, 2012 ▸; Courtade *et al.*, 2016 ▸) and atomic force microscopy (Eibinger *et al.*, 2014 ▸). Transient interactions with natural electron donors may also turn out to be more amenable to NMR spectroscopy (Courtade *et al.*, 2016 ▸) than crystallography, unless the complexes can be stabilized.

In many organisms, there is a tremendous redundancy of LMPOs, in particular in the fungal family AA9. It has been estimated that plant cell wall-degrading fungi have an average of ten AA9 genes (Busk & Lange, 2015 ▸), with some having rather more extreme numbers. We can expect that for AA9 in particular we have as yet to see the full functional and structural diversity, and crystallography will continue to play an important role in this journey of discovery. We have already seen that although initially all LPMOs seemed to be crystalline polysaccharide degraders with flat binding surfaces, we now know that they also can degrade soluble hemicelluloses with a β-1,4-glucan backbone, as does *Nc*AA9_C (Agger *et al.*, 2014 ▸), and there are indications, although no structural characterization, that some members of the family may degrade xylan, alone or when bound to cellulose (Frommhagen *et al.*, 2015 ▸; Kim *et al.*, 2016 ▸). Unusual members of the LPMO family with a missing His brace owing to an N-terminal Arg have been noted in *Phanerochaete chrysosporium* and *Heterobasidion irregulare* (Yakovlev *et al.*, 2012 ▸; Wu *et al.*, 2013 ▸). Recently, there has even been a suggestion that AA11 may play an important role in keratin degradation, although this is not likely to be by attacking keratin itself (Lange *et al.*, 2016 ▸).

Aside from the structural questions, there are number of more biological questions about LPMOs, for example their role in the global carbon cycle and their regulation and role in the regulation of biomass degradation in nature, as well as the exciting prospect of fine-tuning them as tools to turn our garbage into convenient energy. Clearly, a fundamental understanding of the reaction has a role to play here, as suggested by a recent report of light activation of LPMOs using photosynthetic pigments such as chlorophyll (Cannella *et al.*, 2016 ▸), with a staggering effect on their activity.

*Note added in proof.* An additional structure of *Ba*AA10_A has been deposited in the PDB (PDB entry 5iju) after Gregory *et al.* (2016 ▸).

## Figures and Tables

**Table 1:** X-ray crystal structures of LPMOs The key to the footnote markers is given at the end of the table.

					Crystallization and diffraction data				Active site		Specificity			
Family	Organism	Protein name	PDB code	ASU†	Protein concentration and buffer	Crystallization conditions	Space group	Res. (Å)	Element (oxidation)‡	Residues	Substrates	Site of attack	Comments	Reference
AA9	*Lentinus similis*	*Ls*AA9_A	5acf	1	19.2 mg ml^−1^, 0.02 *M* sodium acetate pH 5.5	3.6 *M* NaCl, 0.1 *M* citric acid pH 4.0 (pH 5.5)	*P*4_1_32	1.80	Copper(II)	MeHis1, His78, Tyr164	PASC, cellooligosaccharides	C4	Preincubated with 1 m*M* copper(II) acetate. Cellotriose bound in the active site. Exogenous ligand (Cl^−^) mimicking superoxide.	Frandsen *et al.* (2016 ▸)
AA9	*Lentinus similis*	*Ls*AA9_A	5acg	1	19.2 mg ml^−1^, 0.02 *M* sodium acetate pH 5.5	3.9 *M* NaCl, 0.1 *M* citric acid pH 4.0 (pH 5.5)	*P*4_1_32	1.91	Copper(II)	MeHis1, His78, Tyr164	PASC, cellooligosaccharides	C4	Preincubated with 1 m*M* copper(II) acetate.	Frandsen *et al.* (2016 ▸)
AA9	*Lentinus similis*	*Ls*AA9_A	5ach	1	8.5 mg ml^−1^, 0.02 *M* sodium acetate pH 5.5	3.0 *M* NaCl, 0.1 *M* citric acid pH 3.5 (pH 5.5)	*P*4_1_32	1.28	Copper(II)/copper(I) 0.9	MeHis1, His78, Tyr164	PASC, cellooligosaccharides	C4	Preincubated with 1 m*M* copper(II) acetate.	Frandsen *et al.* (2016 ▸)
AA9	*Lentinus similis*	*Ls*AA9_A	5aci	1	19.2 mg ml^−1^, 0.02 *M* sodium acetate pH 5.5	3.6 *M* NaCl, 0.1 *M* citric acid pH 4.0 (pH 5.5)	*P*4_1_32	1.75	Copper(II)/copper(I)	MeHis1, His78, Tyr164	PASC, cellooligosaccharides	C4	Preincubated with 1 m*M* copper(II) acetate. Cellohexaose bound in the active site.	Frandsen *et al.* (2016 ▸)
AA9	*Lentinus similis*	*Ls*AA9_A	5acj	1	19.2 mg ml^−1^, 0.02 *M* sodium acetate pH 5.5	3.0 *M* NaCl, 0.1 *M* citric acid pH 3.5 (pH 5.5)	*P*4_1_32	1.70	Copper(I)	MeHis1, His78, Tyr164	PASC, cellooligosaccharides	C4	Preincubated with 1 m*M* copper(II) acetate. Cellotriose bound in the active site.	Frandsen *et al.* (2016 ▸)
AA9	*Neurospora crassa*	*Nc*AA9_D, *Nc*LPMO9D, PMO-2, NCU01050, GH61-4	4eir	2	0.15 *M* NaCl, 0.010 *M* Tris pH 8.5	PEG 3350 (pH 6.7)	*P*2_1_	1.10	Copper(II)/copper(I)	MeHis1, His84, Tyr168	PASC	C4	Proposed O_2_ molecule near active-site axial position.	Li *et al.* (2012 ▸), Phillips *et al.* (2011 ▸)
AA9	*Neurospora crassa*	*Nc*AA9_F, *Nc*LPMO9F, PMO-03328, NCU03328, GH61-6	4qi8	2	—	0.2 *M* NH_4_NO_3_, 20%(*w*/*v*) PEG 3350 pH 7.0	*P*2_1_2_1_2	1.10	Copper(II)	His1, His72, Tyr157	PASC	C1	Regiospecificity in Vu, Beeson, Phillips *et al.* (2014 ▸).	Tan *et al.* (2015 ▸), Phillips *et al.* (2011 ▸)
AA9	*Neurospora crassa*	*Nc*AA9_M, *Nc*LPMO9M, *Nc*PMO-3, PMO-3, NCU07898, GH61-13	4eis	2	104 mg ml^−1^, 0.010 *M* Tris pH 8.5	0.1 *M* NaCl, 0.010 *M* Tris pH 8.5	*P*2_1_	1.37	Copper(I)	MeHis1, His82, Tyr171	PASC	C1/C4	Proposed peroxide ion near active site. Tyr24 oxidation.	Li *et al.* (2012 ▸), Phillips *et al.* (2011 ▸)
AA9	*Neurospora crassa*	*Nc*AA9_C, *Nc*LPMO9C, NCU02916, PMO-02916, GH61-3	4d7u	2	1.4 mg ml^−1^	0.2 *M* NH_4_ citrate, 20%(*w*/*v*) PEG 3350 pH 5.1	*P*2_1_	1.56	Copper(I)	His1, His83, Tyr166	PASC, cellooligosaccharides, xyloglucan, glucomannan, β-glucan	C4	—	Borisova *et al.* (2015 ▸)
AA9	*Neurospora crassa*	*Nc*AA9_C, *Nc*LPMO9C, NCU02916, PMO-02916, GH61-3	4d7v	2	1.4 mg ml^−1^	0.2 *M* zinc(II) acetate, 17.5/20.0%(*w*/*v*) PEG 3350 pH 8.0	*P*2_1_	1.90	Zinc(II)	His1, His83, Tyr166	PASC, cellooligosaccharides, xyloglucan, glucomannan, β-glucan	C4	—	Borisova *et al.* (2015 ▸)
AA9	*Phanerochaete chrysosporium*	*Pc*AA9_D, *Pc*LPMO9D, *Pc*GH61D, GH61D	4b5q	2	12 mg ml^−1^, 0.01 *M* sodium acetate pH 5.0	2.1 *M* malic acid (racemic) pH 7.0	*C*2	1.75	Copper(I)	His1, His76, Tyr160	PASC, Avicel	C1	—	Wu *et al.* (2013 ▸)
AA9	*Thermoascus aurantiacus*	*Ta*AA9_A, *Ta*AA9A, *Ta*GH61, *Ta*GH61A, *Ta*LPMO9A	3zud	1	15 mg ml^−1^, 0.02 *M* sodium acetate pH 5.5	0.2 *M* NaCl, 0.1 *M* HEPES pH 7.5, 25%(*w*/*v*) PEG 3350	*P*2_1_	1.25	Copper(II)/copper(I); *A*, 0.6; *B*, 0.15	MeHis1, His86, Tyr175	PASC, PCS	C1/C4	Crystal soaked in 10 m*M* copper(II) nitrate.	Quinlan *et al.* (2011 ▸)
AA9	*Thermoascus aurantiacus*	*Ta*AA9_A, *Ta*AA9A, *Ta*GH61, *Ta*GH61A, *Ta*LPMO9A	2yet	2	15 mg ml^−1^, 0.02 *M* sodium acetate pH 5.5	0.2 *M* NaCl, 0.1 *M* HEPES pH 8.0, 25%(*w*/*v*) PEG 3350	*P*2_1_	1.50	Copper 0.2	MeHis1, His86, Tyr175	PASC, PCS	C1/C4		Quinlan *et al.* (2011 ▸)
AA9	*Thielavia terrestris*	*Tt*AA9_E, *Tt*GH61E, GH61E, 131562	3eii	4	3.1 mg ml^−1^ (pH 7.6–5.0)	1.6 *M* MgSO_4_, 0.1 *M* MES pH 6.5	*F*23	2.25	Zinc(II)	His1, His68, Tyr153	PASC, Avicel	C1	Crystal soaked in 1.8 *M* ZnSO_4_, cacodylate pH 6.5. Regiospecificity inferred from Cannella *et al.* (2016 ▸).	Harris *et al.* (2010 ▸)
AA9	*Thielavia terrestris*	*Tt*AA9_E, TtGH61E, GH61E, 131562	3eja	4	3.1 mg ml^−1^ (pH 7.6–5.0)	1.6 *M* MgSO_4_, 0.1 *M* MES pH 6.5	*F*23	1.90	Magnesium(II)	His1, His68, Tyr153	PASC, Avicel	C1	—	Harris *et al.* (2010 ▸)
AA9	*Trichoderma reesei*	*Hj*AA9_B, *Hj*GH61B, GH61B, Cel61B, EG7, *Tr*AA9_B	2vtc	2	2.2 mg ml^−1^, 0.02 *M* sodium phosphate pH 6.8	15–20%(*w*/*v*) PEG 2000, 0.1 *M* Tris pH 8.4, 0.010 *M* NiCl_2_	*P*6_5_	1.60	Nickel(II)	His1, His89, Tyr176	Cellulose	N/D	—	Karkehabadi *et al.* (2008 ▸)
AA10	*Bacillus amyloliquefaciens*	*Ba*AA10_A, *Ba*CBM33, ChbB, Rbam17540	2yow	2	7.0 mg ml^−1^, 0.02 *M* sodium acetate pH 5.0, 0.25 *M* NaCl	0.1 *M* MMT pH 4.0, 25%(*w*/*v*) PEG 1500	*P*2_1_2_1_2	1.80	—	His28, His125, Phe196	N/D	N/D	Likely to be active on chitin.	Hemsworth, Davies *et al.* (2013 ▸)
AA10	*Bacillus amyloliquefaciens*	*Ba*AA10_A, *Ba*CBM33, ChbB, Rbam17540	2yox	2	7.0 mg ml^−1^, 0.02 *M* sodium acetate pH 5.0, 0.25 *M* NaCl	0.1 *M* MMT pH 4.0, 25%(*w*/*v*) PEG 1500	*P*2_1_	1.90	Copper(I)	His28, His125, Phe196	N/D	N/D	1 m*M* copper(II) nitrate added to sample.	Hemsworth, Davies *et al.* (2013 ▸)
AA10	*Bacillus amyloliquefaciens*	*Ba*AA10_A, *Ba*CBM33, ChbB, Rbam17540	2yoy	2	7.0 mg ml^−1^, 0.02 *M* sodium acetate pH 5.0, 0.25 *M* NaCl	0.1 *M* MMT pH 4.0, 25%(*w*/*v*) PEG 1500	*P*2_1_2_1_2	1.70	Copper(I)	His28, His125, Phe196	N/D	N/D	—	Hemsworth, Davies *et al.* (2013 ▸)
AA10	*Burkholderia pseudomallei*	*Bp*AA10_A	3uam	6	21 mg ml^−1^	0.1 *M* bis-tris propane pH 6.77, 0.2 *M* NaNO_3_, 20.54%(*w*/*v*) PEG 3500	*P*1	2.00	—	His19, His122, Phe205	N/D	N/D	Mentioned in Book *et al.* (2014 ▸).	To be published
AA10	*Cellvibrio japonicus*	*Cj*AA10_A *Cj*LPMO10A, CJA_2191, Cbp33A, Lpmo10A	5fjq	3	9 mg ml^−1^, 0.02 *M* Tris pH 8.0	0.1 *M* sodium acetate pH 5.2, 22%(*w*/*v*) PEG 4000	*C*2	1.85	Copper(I) [copper(II) in chain *B*]	His37, His136, Phe205	α-Chitin, β-chitin	C1	Copper(II)-saturated sample.	Forsberg *et al.* (2016 ▸)
AA10	*Enterococcus faecalis*	*Ef*AA10_A, *Ef*CBM33A, EfaCBM33, EF0362	4a02	1	25 mg ml^−1^, 0.02 *M* Tris pH 8.0	1.0 *M* K/Na tartrate, 0.1 *M* imidazole pH 8.0, 0.2 *M* NaCl	*P*3_2_	0.95	—	His29, His114, Phe185	α-Chitin, β-chitin	C1	—	Vaaje-Kolstad *et al.* (2012 ▸)
AA10	*Enterococcus faecalis*	*Ef*AA10_A, *Ef*CBM33A, EfaCBM33, EF0362	4alc	1	25 mg ml^−1^, 0.02 *M* Tris pH 8.0	0.1 *M* HEPES pH 7.5, 20%(*w*/*v*) PEG 8000	*P*2_1_2_1_2	1.49	Copper(II)	His29, His114, Phe185	α-Chitin, β-chitin	C1	Preincubated with 1 m*M* CuSO_4_.	Gudmundsson (*et al.*, 2014 ▸)
AA10	*Enterococcus faecalis*	*Ef*AA10_A, *Ef*CBM33A, EfaCBM33, EF0362	4ale	1	25 mg ml^−1^, 0.02 *M* Tris pH 8.0	0.1 *M* HEPES pH 7.5, 20%(*w*/*v*) PEG 8000	*P*2_1_2_1_2	1.48	Copper(II)/copper(I)	His29, His114, Phe185	α-Chitin, β-chitin	C1	Preincubated with 1 m*M* CuSO_4_.	Gudmundsson (*et al.*, 2014 ▸)
AA10	*Enterococcus faecalis*	*Ef*AA10_A, *Ef*CBM33A, EfaCBM33, EF0362	4alq	1	25 mg ml^−1^, 0.02 *M* Tris pH 8.0	0.1 *M* HEPES pH 7.5, 20%(*w*/*v*) PEG 8000	*P*2_1_2_1_2	1.48	Copper(II)/copper(I)	His29, His114, Phe185	α-Chitin, β-chitin	C1	Preincubated with 1 m*M* CuSO_4_.	Gudmundsson (*et al.*, 2014 ▸)
AA10	*Enterococcus faecalis*	*Ef*AA10_A, *Ef*CBM33A, EfaCBM33, EF0362	4alr	1	25 mg ml^−1^, 0.02 *M* Tris pH 8.0	0.1 *M* HEPES pH 7.5, 20%(*w*/*v*) PEG 8000	*P*2_1_2_1_2	1.49	Copper(II)/copper(I)	His29, His114, Phe185	α-Chitin, β-chitin	C1	Preincubated with 1 m*M* CuSO_4_.	Gudmundsson (*et al.*, 2014 ▸)
AA10	*Enterococcus faecalis*	*Ef*AA10_A, *Ef*CBM33A, EfaCBM33, EF0362	4als	1	25 mg ml^−1^, 0.02 *M* Tris pH 8.0	0.1 *M* HEPES pH 7.5, 20%(*w*/*v*) PEG 8000	*P*2_1_2_1_2	1.47	Copper(II)/copper(I)	His29, His114, Phe185	α-Chitin, β-chitin	C1	Preincubated with 1 m*M* CuSO_4_.	Gudmundsson (*et al.*, 2014 ▸)
AA10	*Enterococcus faecalis*	*Ef*AA10_A, *Ef*CBM33A, EfaCBM33, EF0362	4alt	1	25 mg ml^−1^, 0.02 *M* Tris pH 8.0	0.1 *M* HEPES pH 7.5, 20%(*w*/*v*) PEG 8000	*P*2_1_2_1_2	1.49	Copper(I)	His29, His114, Phe185	α-Chitin, β-chitin	C1	Preincubated with 1 m*M* CuSO_4_.	Gudmundsson (*et al.*, 2014 ▸)
AA10	*Jonesia denitrificans*	*Jd*AA10_A, *Jd*LPMO10A, Jden_1381	5aa7	2	20 mg ml^−1^, 0.02 *M* Tris pH 8.0	1.9 *M* DL-malic acid pH 7	*P*2_1_2_1_2_1_	1.55	Copper(I)	His32, His109, Phe164	α-Chitin, β-chitin	C1	—	Mekasha *et al.* (2016 ▸)
AA10	*Serratia marcescens*	*Sm*AA10_A, *Sm*LPMO10A, CBP21, Cbp21, Cbp	2bem	3	20 mg ml^−1^, 0.05 *M* Tris pH 8.0	1.26 *M* (NH_4_)_2_SO_4_, 0.1 *M* HEPES pH 7.5	*P*2_1_2_1_2	1.55	Na^2+^	His28, His114, Phe187	α-Chitin, β-chitin	C1	Chain *A* and *B* have no active-site metal.	Vaaje-Kolstad, Houston *et al.*, 2005 ▸)
AA10	*Serratia marcescens*	*Sm*AA10_A, SmLPMO10A, CBP21, Cbp21, Cbp	2ben	2	17.5 mg ml^−1^, 0.05 *M* Tris pH 8.0	20%(*w*/*v*) PEG 8000, 0.1 *M* CHAPS, 0.2 *M* NaCl	*P*3_2_21	1.80	—	His28, His114, Phe187	α-Chitin, β-chitin	C1	Y54A mutant.	Vaaje-Kolstad, Houston *et al.* (2005 ▸)
AA10	*Serratia marcescens*	*Sm*AA10_A, *Sm*LPMO10A, CBP21, Cbp21, Cbp	2lhs	—	0.8–1.2 m*M*, 0.02 *M* K_3_PO_4_, 0.01 *M* NaCl	pH 5.5	—	—	—	His28, His114, Phe187	α-Chitin, β-chitin	C1	NMR structure.	Aachmann *et al.* (2012 ▸)
AA10	*Streptomyces coelicolor*	*Sc*AA10_B, *Sc*LPMO10B, SCO0643, SCF91.03c	4oy6	1	10.3 mg ml^−1^, 0.02 *M* Tris pH 8.0	0.2 *M* zinc acetate, 0.1 *M* sodium cacodylate pH 6.5, 9%(*v*/*v*) 2-propanol	*P*3_1_21	1.29	Copper(II)/copper(I) 0.95	His43, His150, Tyr219	PASC, Avicel, β-chitin	C1/C4 (C1 on chitin)	Soaked in 1–20 m*M* CuCl_2_ (zinc acetate reduced to 0.1 *M*).	Forsberg, Mackenzie *et al.* (2014 ▸)
AA10	*Streptomyces coelicolor*	*Sc*AA10_B, *Sc*LPMO10B, SCO0643, SCF91.03c	4oy8	1	10.3 mg ml^−1^, 0.02 *M* Tris pH 8.0	0.2 *M* zinc acetate, 0.1 *M* sodium cacodylate pH 6.5, 9%(*v*/*v*) 2-propanol	*P*3_1_21	1.40	Zinc(II) 0.8	His43, His150, Tyr219	PASC, Avicel, β-chitin	C1/C4 (C1 on chitin)	—	Forsberg, Mackenzie *et al.* (2014 ▸)
AA10	*Streptomyces coelicolor*	*Sc*AA10_C, *Sc*LPMO10C, LPMO10C, CelS2, SCO1188, SCG11A.19	4oy7	8	9.2 mg ml^−1^, 0.02 *M* Tris pH 8.0	9%(*w*/*v*) PEG 10 000, 0.1 *M* sodium citrate, 0.1 *M* calcium acetate, 5%(*v*/*v*) glycerol	*P*2_1_2_1_2_1_	1.50	Copper(II)/copper(I) 0.5	His35, His144, Phe219	PASC, Avicel	C1	—	Forsberg, Mackenzie *et al.* (2014 ▸)
AA10	*Streptomyces lividans*	*Sl*AA10_E, SliLPMO10E, SLI_3182	5ftz	1	15 mg ml^−1^, 0.01 *M* sodium acetate pH 5.0, 0.15 *M* NaCl	0.1 *M* sodium acetate pH 4.6, 25%(*w*/*v*) PEG 4000	*C*2	1.38	Copper(I) 0.8	His30, His120, Phe193	β-Chitin	C1/(C4)	Definite C1 oxidation Indications of C4 oxidation.	Chaplin *et al.* (2016 ▸)
AA10	*Thermobifida fusca*	*Tf*AA10_A, *Tf*LPMO10A, E7, Tfu_1268	4gbo	2	—	0.1 *M* HEPES pH 7.5, 4.3 *M* NaCl	*P*3_2_21	2.00	Copper(I) 0.5	His37, His144, Tyr213	PASC, Avicel, β-chitin	C1/C4 (C1 on chitin)	Regiospecificity in Forsberg, Mackenzie *et al.* (2014 ▸).	To be published
AA10	*Vibrio cholerae*	*Vc*AA10_B, VCA0811, *Vc*GbpA, GbpA	2xwx	2	2 mg ml^−1^, 0.02 *M* Tris pH 7.5	0.2 *M* Mg(HCO_3_)_2_, 50%(*w*/*v*) PEG 3350, 3.33%(*w*/*v*) D-sorbitol pH 7.5	*P*2_1_	1.80	—	His24, His121, Phe193	N/D	N/D	—	Wong *et al.* (2012 ▸)
AA10	*Anomala cuprea entomopoxvirus*	Fusolin (ACV034)	4yn1	1	—	*In vivo* crystallization (pH 7.0)	*P*4_1_2_1_2	1.90	—	His1, His142, Phe225	N/D	N/D	Intracellular fusolin microcrystals from EPV-infected larvae of *A. cuprea* moths.	Chiu *et al.* (2015 ▸)
AA10	Unidentified entomopoxvirus	Fusolin (partial)	4yn2	1	—	*In vivo* crystallization (pH 7.0)	*P*4_1_2_1_2	2.02	Zinc(II)	His1, His222	N/D	N/D	Intracellular fusolin microcrystals from EPV-infected larvae of *Wiseana* spp. moths.	Chiu *et al.* (2015 ▸)
AA10	Unidentified entomopoxvirus (*Melolontha melolontha entomopoxvirus*)	Fusolin	4ow5	1	—	*In vivo* crystallization	*P*4_1_2_1_2	1.90	(H_2_O)	His1, His142, Phe225	N/D	N/D	Active-site water molecule may be a low-occupied metal ion. Chitin-binding domain. Mutations: G25D, H192N, I351N, I352H, Q353T, D354G.	Chiu *et al.* (2015 ▸)
AA10	Unidentified entomopoxvirus (*Melolontha melolontha entomopoxvirus*)	Fusolin	4x27	1	—	*In vivo* crystallization	*P*4_1_2_1_2	2.40	Copper(II) 0.79	His1, His142, Phe225	N/D	N/D	Soaked with CuSO_4_.	Chiu *et al.* (2015 ▸)
AA10	Unidentified entomopoxvirus (*Melolontha melolontha entomopoxvirus*)	Fusolin	4x29	1	—	*In vivo* crystallization	*P*4_1_2_1_2	2.41	Zinc(II)	His1, His142, Phe225	N/D	N/D	Soaked with ZnSO_4_.	Chiu *et al.* (2015 ▸)
AA11	*Aspergillus oryzae*	*Ao*AA11 *Ao*(LPMO11) (AO090102000501)	4mah	1	25 mg ml^−1^, 0.02 *M* sodium acetate pH 5.0	0.01 *M* ZnCl_2_, 0.1 *M* MES pH 6.0, 20%(*w*/*v*) PEG 6000	*P*2_1_2_1_2_1_	1.55	Zinc(II)	His1, His60, Tyr140	β-Chitin	C1	—	Hemsworth *et al.* (2014 ▸)
AA11	*Aspergillus oryzae*	*Ao*AA11 *Ao*(LPMO11) (AO090102000501)	4mai	1	25 mg ml^−1^, 0.02 *M* sodium acetate pH 5.0	0.01 *M* ZnCl_2_, 0.1 *M* MES pH 6.0, 20%(*w*/*v*) PEG 6000	*P*2_1_2_1_2_1_	1.40	Copper(I)	His1, His60, Tyr140	β-Chitin	C1	Soaked in 2 m*M* CuCl_2_.	Hemsworth *et al.* (2014 ▸)
AA13	*Aspergillus oryzae*	*Ao*AA13 (AO090701000246) (AOR_1_454114)	4opb	1	3 mg ml^−1^, 0.02 *M* MES pH 6.0, 0.125 *M* NaCl	0.14 *M* CaCl_2_, 0.07 *M* sodium acetate pH 4.6, 14%(*v*/*v*) 2-propanol, 30%(*v*/*v*) glycerol (+ seed stock)	*P*2_1_2_1_2_1_	1.55	Copper(I)	MeHis1, His91, Tyr224	N/D (starch)	N/D (C1)	Enzymes with 70–72% sequence identity [*An*AA13 in Lo Leggio *et al.* (2015 ▸) and* Nc*AA13 in Vu, Beeson, Phillips *et al.* (2014 ▸)] release C1-oxidized products from starch-related substrates.	Lo Leggio *et al.* (2015 ▸)

† Number of molecules in the asymmetric unit.

‡ The criteria for assigning a copper(II) or copper(I) state were informed by structures where both states have been characterized (Gudmundsson *et al.*, 2014 ▸). The electron density of the equatorial exogenous ligand to the copper (from weighted 2*F*_obs_ − *F*_calc_) should be more than 2σ with more than 0.5 occupancy and a distance to the copper of less than 2.4 Å, with similar criteria applying to the exogenous axial ligand, although with a distance of 2.8 Å. In structures where a distorted geometry is observed because of significant steric effects (most AA10 members), structures with a single exogenous ligand within 2.5 Å distance are taken as copper(II). The occupancy of the metal is 1.00 if no other value is indicated. If there is significant metal-site disorder with characteristics that could fit both states, the site is described as copper(II)/copper(I). When the copper occupancy was lower than 0.5, no oxidation state was assigned.

## References

[bb1] Aachmann, F. L., Sørlie, M., Skjåk-Braek, G., Eijsink, V. G. H. & Vaaje-Kolstad, G. (2012). *Proc. Natl Acad. Sci. USA*, **109**, 18779–18784. 10.1073/pnas.1208822109 PMC3503203 23112164

[bb2] Agger, J. W., Isaksen, T., Várnai, A., Vidal-Melgosa, S., Willats, W. G., Ludwig, R., Horn, S. J., Eijsink, V. G. H. & Westereng, B. (2014). *Proc. Natl Acad. Sci. USA*, **111**, 6287–6292. 10.1073/pnas.1323629111 PMC4035949 24733907

[bb3] Bacik, J.-P., Mekasha, S., Forsberg, Z., Kovalevsky, A., Nix, J. C., Cuneo, M. J., Coates, L., Vaaje-Kolstad, G., Chen, J. C.-H., Eijsink, V. G. H. & Unkefer, C. J. (2015). *Acta Cryst.* F**71**, 1448–1452. 10.1107/S2053230X15019743 PMC4631597 26527275

[bb4] Bayer, E. A., Lamed, R. & Himmel, M. E. (2007). *Curr. Opin. Biotechnol.* **18**, 237–245. 10.1016/j.copbio.2007.04.004 17462879

[bb5] Beeson, W. T., Phillips, C. M., Cate, J. H. & Marletta, M. A. (2012). *J. Am. Chem. Soc.* **134**, 890–892. 10.1021/ja210657t 22188218

[bb6] Beeson, W. T., Vu, V. V., Span, E. A., Phillips, C. M. & Marletta, M. A. (2015). *Annu. Rev. Biochem.* **84**, 923–946. 10.1146/annurev-biochem-060614-034439 25784051

[bb7] Béguin, P. (1990). *Annu. Rev. Microbiol.* **44**, 219–248. 10.1146/annurev.mi.44.100190.001251 2252383

[bb8] Bennati-Granier, C., Garajova, S., Champion, C., Grisel, S., Haon, M., Zhou, S., Fanuel, M., Ropartz, D., Rogniaux, H., Gimbert, I., Record, E. & Berrin, J.-G. (2015). *Biotechnol. Biofuels*, **8**, 90. 10.1186/s13068-015-0274-3 PMC4487207 26136828

[bb9] Blake, C. C. F., Koenig, D. F., Mair, G. A., North, A. C. T., Phillips, D. C. & Sarma, V. R. (1965). *Nature (London)*, **206**, 757–761. 10.1038/206757a0 5891407

[bb10] Book, A. J., Yennamalli, R. M., Takasuka, T. E., Currie, C. R., Phillips, G. N. & Fox, B. G. (2014). *Biotechnol. Biofuels*, **7**, 109. 10.1186/1754-6834-7-109 PMC4144037 25161697

[bb11] Boraston, A. B., Bolam, D. N., Gilbert, H. J. & Davies, G. J. (2004). *Biochem. J.* **382**, 769–781. 10.1042/BJ20040892 PMC1133952 15214846

[bb12] Borisova, A. S., Isaksen, T., Dimarogona, M., Kognole, A. A., Mathiesen, G., Várnai, A., Røhr, Å. K., Payne, C. M., Sørlie, M., Sandgren, M. & Eijsink, V. G. H. (2015). *J. Biol. Chem.* **290**, 22955–22969. 10.1074/jbc.M115.660183 PMC4645601 26178376

[bb13] Bornscheuer, U., Buchholz, K. & Seibel, J. (2014). *Angew. Chem. Int. Ed.* **53**, 10876–10893. 10.1002/anie.201309953 25136976

[bb14] Brown, K., Harris, P., Zaretsky, E., Re, E., Vlasenko, E., Mcfarland, K. & Lopez, D. L. A. (2006). Patent WO2005074647.

[bb15] Busk, P. K. & Lange, L. (2015). *BMC Genomics*, **16**, 368. 10.1186/s12864-015-1601-6 PMC4424831 25956378

[bb16] Cannella, D., Möllers, K. B., Frigaard, N. U., Jensen, P. E., Bjerrum, M. J., Johansen, K. S. & Felby, C. (2016). *Nat. Commun.* **7**, 11134. 10.1038/ncomms11134 PMC4822002 27041218

[bb17] Carpita, N. C. & Gibeaut, D. M. (1993). *Plant J.* **3**, 1–30. 10.1111/j.1365-313x.1993.tb00007.x 8401598

[bb18] Chaplin, A. K., Wilson, M. T., Hough, M. A., Svistunenko, D. A., Hemsworth, G. R., Walton, P. H., Vijgenboom, E. & Worrall, J. A. R. (2016). *J. Biol. Chem.* **291**, 12838–12850. 10.1074/jbc.M116.722447 PMC4933455 27129229

[bb19] Chen, P. & Solomon, E. I. (2004). *J. Am. Chem. Soc.* **126**, 4991–5000. 10.1021/ja031564g 15080705

[bb20] Chiu, E., Hijnen, M., Bunker, R. D., Boudes, M., Rajendran, C., Aizel, K., Oliéric, V., Schulze-Briese, C., Mitsuhashi, W., Young, V., Ward, V. K., Bergoin, M., Metcalf, P. & Coulibaly, F. (2015). *Proc. Natl Acad. Sci. USA*, **112**, 3973–3978. 10.1073/pnas.1418798112 PMC4386404 25787255

[bb21] Courtade, G., Wimmer, R., Røhr, A. K., Preims, M., Felice, A. K. G., Dimarogona, M., Vaaje-Kolstad, G., Sørlie, M., Sandgren, M., Ludwig, R., Eijsink, V. G. H. & Aachmann, F. L. (2016). *Proc. Natl Acad. Sci. USA*, **113**, 5922–5927. 10.1073/pnas.1602566113 PMC4889390 27152023

[bb22] Cragg, S. M., Beckham, G. T., Bruce, N. C., Bugg, T. D. H., Distel, D. L., Dupree, P., Etxabe, A. G., Goodell, B. S., Jellison, J., McGeehan, J. E., McQueen-Mason, S. J., Schnorr, K., Walton, P. H., Watts, J. E. M. & Zimmer, M. (2015). *Curr. Opin. Chem. Biol.* **29**, 108–119. 10.1016/j.cbpa.2015.10.018 PMC7571853 26583519

[bb23] Crichton, R. R. (2012). *Biological Inorganic Chemistry: A New Introduction to Molecular Structure and Function*, 2nd ed. Amsterdam: Elsevier.

[bb24] Crouch, L. I., Labourel, A., Walton, P. H., Davies, G. J. & Gilbert, H. J. (2016). *J. Biol. Chem.* **291**, 7439–7449. 10.1074/jbc.M115.702365 PMC4817175 26801613

[bb25] Davies, G. J., Mackenzie, L., Varrot, A., Dauter, M., Brzozowski, A. M., Schülein, M. & Withers, S. G. (1998). *Biochemistry*, **37**, 11707–11713. 10.1021/bi981315i 9718293

[bb26] Davies, G. J., Wilson, K. S. & Henrissat, B. (1997). *Biochem. J.* **321**, 557–559. 10.1042/bj3210557 PMC1218105 9020895

[bb27] Dhar, D. & Tolman, W. B. (2015). *J. Am. Chem. Soc.* **137**, 1322–1329. 10.1021/ja512014z PMC4311965 25581555

[bb28] Dimarogona, M., Topakas, E., Olsson, L. & Christakopoulos, P. (2012). *Bioresour. Technol.* **110**, 480–487. 10.1016/j.biortech.2012.01.116 22342036

[bb29] Doan, N. & Gettins, P. W. (2007). *Biochem. J.* **407**, 23–30. 10.1042/BJ20070764 PMC2267405 17608619

[bb30] Donoghue, P. J., Tehranchi, J., Cramer, C. J., Sarangi, R., Solomon, E. I. & Tolman, W. B. (2011). *J. Am. Chem. Soc.* **133**, 17602–17605. 10.1021/ja207882h PMC3213683 22004091

[bb31] Eibinger, M., Ganner, T., Bubner, P., Rošker, S., Kracher, D., Haltrich, D., Ludwig, R., Plank, H. & Nidetzky, B. (2014). *J. Biol. Chem.* **289**, 35929–35938. 10.1074/jbc.M114.602227 PMC4276861 25361767

[bb32] Eriksson, K.-E., Pettersson, B. & Westermark, U. (1974). *FEBS Lett.* **49**, 282–285. 10.1016/0014-5793(74)80531-4 4474960

[bb33] Fleming, A. (1922). *Proc. R. Soc. B: Biol. Sci.* **93**, 306–317.

[bb34] Floudas, D. *et al.* (2012). *Science*, **336**, 1715–1719. 10.1126/science.1221748 22745431

[bb35] Floudas, D. *et al.* (2015). *Fungal Genet. Biol.* **76**, 78–92. 10.1016/j.fgb.2015.02.002 PMC4399860 25683379

[bb36] Forsberg, Z., Mackenzie, A. K., Sørlie, M., Røhr, Å. K., Helland, R., Arvai, A. S., Vaaje-Kolstad, G. & Eijsink, V. G. H. (2014). *Proc. Natl Acad. Sci. USA*, **111**, 8446–8451. 10.1073/pnas.1402771111 PMC4060697 24912171

[bb37] Forsberg, Z., Nelson, C. E., Dalhus, B., Mekasha, S., Loose, J. S. M., Crouch, L. I., Røhr, A. K., Gardner, J. G., Eijsink, V. G. H. & Vaaje-Kolstad, G. (2016). *J. Biol. Chem.* **291**, 7300–7312. 10.1074/jbc.M115.700161 PMC4817163 26858252

[bb38] Forsberg, Z., Røhr, Å. K., Mekasha, S., Andersson, K. K., Eijsink, V. G. H., Vaaje-Kolstad, G. & Sørlie, M. (2014). *Biochemistry*, **53**, 1647–1656. 10.1021/bi5000433 24559135

[bb39] Forsberg, Z., Vaaje-Kolstad, G., Westereng, B., Bunaes, A. C., Stenstrøm, Y., MacKenzie, A., Sørlie, M., Horn, S. J. & Eijsink, V. G. H. (2011). *Protein Sci.* **20**, 1479–1483. 10.1002/pro.689 PMC3190143 21748815

[bb40] Frandsen, K. E. *et al.* (2016). *Nat. Chem. Biol.* **12**, 298–303. 10.1038/nchembio.2029 PMC4817220 26928935

[bb41] Frederiksen, R. F., Paspaliari, D. K., Larsen, T., Storgaard, B. G., Larsen, M. H., Ingmer, H., Palcic, M. M. & Leisner, J. J. (2013). *Microbiology*, **159**, 833–847. 10.1099/mic.0.051839-0 23519157

[bb42] Frommhagen, M., Sforza, S., Westphal, A. H., Visser, J., Hinz, S. W. A., Koetsier, M. J., van Berkel, W. J. H., Gruppen, H. & Kabel, M. A. (2015). *Biotechnol. Biofuels*, **8**, 101. 10.1186/s13068-015-0284-1 PMC4504452 26185526

[bb43] Fuchs, R. L., McPherson, S. A. & Drahos, D. J. (1986). *Appl. Environ. Microbiol.* **51**, 504–509. 10.1128/aem.51.3.504-509.1986 PMC238909 16347012

[bb44] Gagnon, N. & Tolman, W. B. (2015). *Acc. Chem. Res.* **48**, 2126–2131. 10.1021/acs.accounts.5b00169 PMC4856291 26075312

[bb45] Garajova, S., Mathieu, Y., Beccia, M. R., Bennati-Granier, C., Biaso, F., Fanuel, M., Ropartz, D., Guigliarelli, B., Record, E., Rogniaux, H., Henrissat, B. & Berrin, J.-G. (2016). *Sci. Rep.* **6**, 28276. 10.1038/srep28276 PMC4911613 27312718

[bb46] Gibson, D. M., King, B. C., Hayes, M. L. & Bergstrom, G. C. (2011). *Curr. Opin. Microbiol.* **14**, 264–270. 10.1016/j.mib.2011.04.002 21536481

[bb47] Glass, N. L., Schmoll, M., Cate, J. H. D. & Coradetti, S. (2013). *Annu. Rev. Microbiol.* **67**, 477–498. 10.1146/annurev-micro-092611-150044 23808333

[bb123] Gregory, R. C., Hemsworth, G. R., Turkenburg, J. P., Hart, S. J., Walton, P. H. & Davies, G. J. (2016). *Dalton Trans.* 10.1039/c6dt02793h. 10.1039/c6dt02793h 27722375

[bb48] Gudmundsson, M., Kim, S., Wu, M., Ishida, T., Momeni, M. H., Vaaje-Kolstad, G., Lundberg, D., Royant, A., Stahlberg, J., Eijsink, V. G. H., Beckham, G. T. & Sandgren, M. (2014). *J. Biol. Chem.* **289**, 18782–18792. 10.1074/jbc.M114.563494 PMC4081921 24828494

[bb49] Guerriero, G., Hausman, J. F., Strauss, J., Ertan, H. & Siddiqui, K. S. (2016). *Eng. Life Sci.* **16**, 1–16.

[bb50] Harris, P. V., Welner, D., McFarland, K. C., Re, E., Navarro Poulsen, J. C., Brown, K., Salbo, R., Ding, H., Vlasenko, E., Merino, S., Xu, F., Cherry, J., Larsen, S. & Lo Leggio, L. (2010). *Biochemistry*, **49**, 3305–3316. 10.1021/bi100009p 20230050

[bb51] Harris, P. V., Xu, F., Kreel, N. E., Kang, C. & Fukuyama, S. (2014). *Curr. Opin. Chem. Biol.* **19**, 162–170. 10.1016/j.cbpa.2014.02.015 24681544

[bb52] Hayes, M., Carney, B., Slater, J. & Brück, W. (2008). *Biotechnol. J.* **3**, 878–889. 10.1002/biot.200800027 18320569

[bb53] Hellemond, E. W. van, Leferink, N. G. H., Heuts, D., Fraaije, M. W. & van Berkel, W. J. H. (2006). *Adv. Appl. Microbiol.*, **60**, 17–54. 10.1016/S0065-2164(06)60002-6 17157632

[bb54] Hemsworth, G. R., Davies, G. J. & Walton, P. H. (2013). *Curr. Opin. Struct. Biol.* **23**, 660–668. 10.1016/j.sbi.2013.05.006 23769965

[bb55] Hemsworth, G. R., Henrissat, B., Davies, G. J. & Walton, P. H. (2014). *Nat. Chem. Biol.* **10**, 122–126. 10.1038/nchembio.1417 PMC4274766 24362702

[bb56] Hemsworth, G. R., Johnston, E. M., Davies, G. J. & Walton, P. H. (2015). *Trends Biotechnol.* **33**, 747–761. 10.1016/j.tibtech.2015.09.006 26472212

[bb57] Hemsworth, G. R., Taylor, E. J., Kim, R. Q., Gregory, R. C., Lewis, S. J., Turkenburg, J. P., Parkin, A., Davies, G. J. & Walton, P. H. (2013). *J. Am. Chem. Soc.* **135**, 6069–6077. 10.1021/ja402106e PMC3636778 23540833

[bb58] Henrissat, B. (1991). *Biochem. J.* **280**, 309–316. 10.1042/bj2800309 PMC1130547 1747104

[bb59] Henrissat, B., Claeyssens, M., Tomme, P., Lemesle, L. & Mornon, J.-P. (1989). *Gene*, **81**, 83–95. 10.1016/0378-1119(89)90339-9 2806912

[bb60] Henrissat, B. & Davies, G. (1997). *Curr. Opin. Struct. Biol.* **7**, 637–644. 10.1016/s0959-440x(97)80072-3 9345621

[bb61] Holm, L., Kääriäinen, S., Rosenström, P. & Schenkel, A. (2008). *Bioinformatics*, **24**, 2780–2781. 10.1093/bioinformatics/btn507 PMC2639270 18818215

[bb62] Horn, S. J., Vaaje-Kolstad, G., Westereng, B. & Eijsink, V. G. H. (2012). *Biotechnol. Biofuels*, **5**, 45. 10.1186/1754-6834-5-45 PMC3492096 22747961

[bb63] Isaksen, T., Westereng, B., Aachmann, F. L., Agger, J. W., Kracher, D., Kittl, R., Ludwig, R., Haltrich, D., Eijsink, V. G. H. & Horn, S. J. (2014). *J. Biol. Chem.* **289**, 2632–2642. 10.1074/jbc.M113.530196 PMC3908397 24324265

[bb64] Itoh, S. (2006). *Curr. Opin. Chem. Biol.* **10**, 115–122. 10.1016/j.cbpa.2006.02.012 16504568

[bb65] Itzstein, M. von & Thomson, R. (2009). *Antiviral Strategies*, edited by H.-G. Krausslich & R. Bartenschlager, pp. 111–154. Berlin, Heidelberg: Springer-Verlag.

[bb66] Johansen, K. S. (2016*a*). *Biochem. Soc. Trans.* **44**, 143–149. 10.1042/BST20150204 26862199

[bb67] Johansen, K. S. (2016*b*). *Trends Plant Sci.*, 10.1016/j.tplants.2016.07.012.

[bb68] Kamachi, T., Kihara, N., Shiota, Y. & Yoshizawa, K. (2005). *Inorg. Chem.* **44**, 4226–4236. 10.1021/ic048477p 15934751

[bb69] Karkehabadi, S., Hansson, H., Kim, S., Piens, K., Mitchinson, C. & Sandgren, M. (2008). *J. Mol. Biol.* **383**, 144–154. 10.1016/j.jmb.2008.08.016 18723026

[bb70] Karlsson, J., Saloheimo, M., Siika-Aho, M., Tenkanen, M., Penttilä, M. & Tjerneld, F. (2001). *Eur. J. Biochem.* **268**, 6498–6507. 10.1046/j.0014-2956.2001.02605.x 11737205

[bb71] Kim, S., Ståhlberg, J., Sandgren, M., Paton, R. S. & Beckham, G. T. (2014). *Proc. Natl Acad. Sci. USA*, **111**, 149–154. 10.1073/pnas.1316609111 PMC3890868 24344312

[bb72] Kim, I. J., Youn, H. J. & Kim, K. H. (2016). *Process Biochem.*, 10.1016/j.procbio.2016.06.017.

[bb73] Kjaergaard, C. H., Qayyum, M. F., Wong, S. D., Xu, F., Hemsworth, G. R., Walton, D. J., Young, N. A., Davies, G. J., Walton, P. H., Johansen, K. S., Hodgson, K. O., Hedman, B. & Solomon, E. I. (2014). *Proc. Natl Acad. Sci. USA*, **111**, 8797–8802. 10.1073/pnas.1408115111 PMC4066490 24889637

[bb74] Klinman, J. P. (2006). *J. Biol. Chem.* **281**, 3013–3016. 10.1074/jbc.R500011200 16301310

[bb75] Kohler, A. *et al.* (2015). *Nat. Genet.* **47**, 410–415. 10.1038/ng.3223 25706625

[bb76] Kracher, D., Scheiblbrandner, S., Felice, A. K. G., Breslmayr, E., Preims, M., Ludwicka, K., Haltrich, D., Eijsink, V. G. H. & Ludwig, R. (2016). *Science*, **352**, 1098–1101. 10.1126/science.aaf3165 27127235

[bb77] Lange, L., Huang, Y. H. & Busk, P. K. (2016). *Appl. Microbiol. Biotechnol.* **100**, 2083–2096. 10.1007/s00253-015-7262-1 PMC4756042 26754820

[bb78] Langston, J. A., Shaghasi, T., Abbate, E., Xu, F., Vlasenko, E. & Sweeney, M. D. (2011). *Appl. Environ. Microbiol.* **77**, 7007–7015. 10.1128/AEM.05815-11 PMC3187118 21821740

[bb79] Levasseur, A., Drula, E., Lombard, V., Coutinho, P. M. & Henrissat, B. (2013). *Biotechnol. Biofuels*, **6**, 41. 10.1186/1754-6834-6-41 PMC3620520 23514094

[bb80] Li, X., Beeson, W. T. IV, Phillips, C. M., Marletta, M. A. & Cate, J. H. D. (2012). *Structure*, **20**, 1051–1061. 10.1016/j.str.2012.04.002 PMC3753108 22578542

[bb81] Lo Leggio, L. *et al.* (2015). *Nat. Commun.* **6**, 5961. 10.1038/ncomms6961 PMC4338556 25608804

[bb82] Lo Leggio, L., Welner, D. & De Maria, L. (2012). *Comput. Struct. Biotechnol. J.* **2**, e201209019. 10.5936/csbj.201209019 PMC3962118 24688660

[bb83] Lombard, V., Golaconda Ramulu, H., Drula, E., Coutinho, P. M. & Henrissat, B. (2014). *Nucleic Acids Res.* **42**, D490–D495. 10.1093/nar/gkt1178 PMC3965031 24270786

[bb84] Loose, J. S. M., Forsberg, Z., Fraaije, M. W., Eijsink, V. G. H. & Vaaje-Kolstad, G. (2014). *FEBS Lett.* **588**, 3435–3440. 10.1016/j.febslet.2014.07.036 25109775

[bb85] Mekasha, S., Forsberg, Z., Dalhus, B., Bacik, J.-P., Choudhary, S., Schmidt-Dannert, C., Vaaje-Kolstad, G. & Eijsink, V. G. H. (2016). *FEBS Lett.* **590**, 34–42. 10.1002/1873-3468.12025 26763108

[bb86] Paspaliari, D. K., Loose, J. S. M., Larsen, M. H. & Vaaje-Kolstad, G. (2015). *FEBS J.* **282**, 921–936. 10.1111/febs.13191 25565565

[bb87] Pérez, S. & Bertoft, E. (2010). *Starch*, **62**, 389–420.

[bb88] Phillips, C. M., Beeson, W. T., Cate, J. H. & Marletta, M. A. (2011). *ACS Chem. Biol.* **6**, 1399–1406. 10.1021/cb200351y 22004347

[bb89] Pollegioni, L., Tonin, F. & Rosini, E. (2015). *FEBS J.* **282**, 1190–1213. 10.1111/febs.13224 25649492

[bb90] Quinlan, R. J. *et al.* (2011). *Proc. Natl Acad. Sci. USA*, **108**, 15079–15084.

[bb91] Rytioja, J., Hildén, K., Yuzon, J., Hatakka, A., de Vries, R. P. & Mäkelä, M. R. (2014). *Microbiol. Mol. Biol. Rev.* **78**, 614–649. 10.1128/MMBR.00035-14 PMC4248655 25428937

[bb92] Shah, F. *et al.* (2016). *New Phytol.* **209**, 1705–1719. 10.1111/nph.13722 PMC5061094 26527297

[bb93] Shepard, E. M. & Dooley, D. M. (2015). *Acc. Chem. Res.* **48**, 1218–1226. 10.1021/ar500460z 25897668

[bb94] Smith, S. M., Rawat, S., Telser, J., Hoffman, B. M., Stemmler, T. L. & Rosenzweig, A. C. (2011). *Biochemistry*, **50**, 10231–10240. 10.1021/bi200801z PMC3364217 22013879

[bb95] Solomon, E. I., Chen, P., Metz, M., Lee, S. K. & Palmer, A. E. (2001). *Angew. Chem. Int. Ed.* **40**, 4570–4590. 10.1002/1521-3773(20011217)40:24<4570::aid-anie4570>3.0.co;2-4 12404359

[bb96] Solomon, E. I., Heppner, D. E., Johnston, E. M., Ginsbach, J. W., Cirera, J., Qayyum, M., Kieber-Emmons, M. T., Kjaergaard, C. H., Hadt, R. G. & Tian, L. (2014). *Chem. Rev.* **114**, 3659–3853. 10.1021/cr400327t PMC4040215 24588098

[bb97] Span, E. A. & Marletta, M. A. (2015). *Curr. Opin. Struct. Biol.* **35**, 93–99. 10.1016/j.sbi.2015.10.002 26615470

[bb98] Stellato, F. *et al.* (2014). *IUCrJ*, **1**, 204–212. 10.1107/S2052252514010070 PMC4107920 25075341

[bb99] Suzuki, K., Suzuki, M., Taiyoji, M., Nikaidou, N. & Watanabe, T. (1998). *Biosci. Biotechnol. Biochem.* **62**, 128–135. 10.1271/bbb.62.128 9501524

[bb100] Tan, T. C., Kracher, D., Gandini, R., Sygmund, C., Kittl, R., Haltrich, D., Hällberg, B. M., Ludwig, R. & Divne, C. (2015). *Nat. Commun.* **6**, 7542. 10.1038/ncomms8542 PMC4507011 26151670

[bb101] Terwilliger, T. C., DiMaio, F., Read, R. J., Baker, D., Bunkóczi, G., Adams, P. D., Grosse-Kunstleve, R. W., Afonine, P. V. & Echols, N. (2012). *J. Struct. Funct. Genomics*, **13**, 81–90. 10.1007/s10969-012-9129-3 PMC3375004 22418934

[bb102] Vaaje-Kolstad, G., Bøhle, L. A., Gåseidnes, S., Dalhus, B., Bjørås, M., Mathiesen, G. & Eijsink, V. G. H. (2012). *J. Mol. Biol.* **416**, 239–254. 10.1016/j.jmb.2011.12.033 22210154

[bb103] Vaaje-Kolstad, G., Horn, S. J., Sørlie, M. & Eijsink, V. G. H. (2013). *FEBS J.* **280**, 3028–3049. 10.1111/febs.12181 23398882

[bb104] Vaaje-Kolstad, G., Horn, S. J., van Aalten, D. M. F., Synstad, B. & Eijsink, V. G. H. (2005). *J. Biol. Chem.* **280**, 28492–28497. 10.1074/jbc.M504468200 15929981

[bb105] Vaaje-Kolstad, G., Houston, D. R., Riemen, A. H. K., Eijsink, V. G. H. & van Aalten, D. M. F. (2005). *J. Biol. Chem.* **280**, 11313–11319. 10.1074/jbc.M407175200 15590674

[bb106] Vaaje-Kolstad, G., Westereng, B., Horn, S. J., Liu, Z., Zhai, H., Sørlie, M. & Eijsink, V. G. H. (2010). *Science*, **330**, 219–222. 10.1126/science.1192231 20929773

[bb107] Vu, V. V., Beeson, W. T., Phillips, C. M., Cate, J. H. D. & Marletta, M. A. (2014). *J. Am. Chem. Soc.* **136**, 562–565. 10.1021/ja409384b 24350607

[bb108] Vu, V. V., Beeson, W. T., Span, E. A., Farquhar, E. R. & Marletta, M. A. (2014). *Proc. Natl Acad. Sci. USA*, **111**, 13822–13827. 10.1073/pnas.1408090111 PMC4183312 25201969

[bb109] Vu, V. V. & Marletta, M. A. (2016). *Cell. Mol. Life Sci.* **73**, 2809–2919. 10.1007/s00018-016-2251-9 PMC11108391 27170366

[bb110] Walton, P. H. & Davies, G. J. (2016). *Curr. Opin. Chem. Biol.* **31**, 195–207. 10.1016/j.cbpa.2016.04.001 27094791

[bb111] Wei, N., Quarterman, J. & Jin, Y.-S. (2013). *Trends Biotechnol.* **31**, 70–77. 10.1016/j.tibtech.2012.10.009 23245657

[bb112] Welner, D. H., Jensen, M. H., McFarland, K. C., Poulsen, J.-C. N., Otten, H., Salbo, R., Christensen, U., Harris, P. V., Larsen, S. & Borchert, T. (2009). In *Biotechnology of Lignocellulose Degradation and Biomass Utilization – Mie Bioforum 2008.* Tokyo: Ito Print Publishing Division.

[bb113] Westereng, B., Cannella, D., Agger, J. W., Jørgensen, H., Andersen, M. L., Eijsink, V. G. H. & Felby, C. (2015). *Sci. Rep.* **5**, 18561. 10.1038/srep18561 PMC4685257 26686263

[bb114] Westereng, B., Ishida, T., Vaaje-Kolstad, G., Wu, M., Eijsink, V. G. H., Igarashi, K., Samejima, M., Ståhlberg, J., Horn, S. J. & Sandgren, M. (2011). *PLoS One*, **6**, e27807. 10.1371/journal.pone.0027807 PMC3223205 22132148

[bb115] Wong, E., Vaaje-Kolstad, G., Ghosh, A., Hurtado-Guerrero, R., Konarev, P. V., Ibrahim, A. F. M., Svergun, D. I., Eijsink, V. G. H., Chatterjee, N. S. & van Aalten, D. M. F. (2012). *PLoS Pathog.* **8**, e1002373. 10.1371/journal.ppat.1002373 PMC3257281 22253590

[bb116] Wu, M., Beckham, G. T., Larsson, A. M., Ishida, T., Kim, S., Payne, C. M., Himmel, M. E., Crowley, M. F., Horn, S. J., Westereng, B., Igarashi, K., Samejima, M., Ståhlberg, J., Eijsink, V. G. H. & Sandgren, M. (2013). *J. Biol. Chem.* **288**, 12828–12839. 10.1074/jbc.M113.459396 PMC3642327 23525113

[bb117] Yakovlev, I., Vaaje-Kolstad, G., Hietala, A. M., Stefańczyk, E., Solheim, H. & Fossdal, C. G. (2012). *Appl. Microbiol. Biotechnol.* **95**, 979–990. 10.1007/s00253-012-4206-x PMC3405238 22718248

[bb118] Yoder, M. D., Keen, N. T. & Jurnak, F. (1993). *Science*, **260**, 1503–1507. 10.1126/science.8502994 8502994

[bb119] Yoshizawa, K., Kihara, N., Kamachi, T. & Shiota, Y. (2006). *Inorg. Chem.* **45**, 3034–3041. 10.1021/ic0521168 16562959

[bb120] Zeng, Y., Zhao, S., Yang, S. & Ding, S.-Y. (2014). *Curr. Opin. Biotechnol.* **27**, 38–45. 10.1016/j.copbio.2013.09.008 24863895

[bb121] Zhang, L., Koay, M., Maher, M. J., Xiao, Z. & Wedd, A. G. (2006). *J. Am. Chem. Soc.* **128**, 5834–5850. 10.1021/ja058528x 16637653

[bb122] Zheng, H., Chordia, M. D., Cooper, D. R., Chruszcz, M., Müller, P., Sheldrick, G. M. & Minor, W. (2014). *Nat. Protoc.* **9**, 156–170. 10.1038/nprot.2013.172 PMC4410975 24356774

